# A genetic screen identifies BEND3 as a regulator of bivalent gene expression and global DNA methylation

**DOI:** 10.1093/nar/gkad719

**Published:** 2023-08-31

**Authors:** Lounis Yakhou, Anaelle Azogui, Nikhil Gupta, Julien Richard Albert, Fumihito Miura, Laure Ferry, Kosuke Yamaguchi, Sarah Battault, Pierre Therizols, Frédéric Bonhomme, Elouan Bethuel, Arpita Sarkar, Maxim V C Greenberg, Paola B Arimondo, Gael Cristofari, Olivier Kirsh, Takashi Ito, Pierre-Antoine Defossez

**Affiliations:** Université Paris Cité, CNRS, Epigenetics and Cell Fate, F-75013 Paris, France; Université Paris Cité, CNRS, Epigenetics and Cell Fate, F-75013 Paris, France; Université Paris Cité, CNRS, Epigenetics and Cell Fate, F-75013 Paris, France; Université Paris Cité, CNRS, Institut Jacques Monod, F-75013 Paris, France; Department of Biochemistry, Kyushu University Graduate School of Medical Sciences, Fukuoka, Fukuoka 812-8582, Japan; Université Paris Cité, CNRS, Epigenetics and Cell Fate, F-75013 Paris, France; Université Paris Cité, CNRS, Epigenetics and Cell Fate, F-75013 Paris, France; Université Paris Cité, CNRS, Epigenetics and Cell Fate, F-75013 Paris, France; Université Paris Cité, CNRS, Epigenetics and Cell Fate, F-75013 Paris, France; Institut Pasteur, Université Paris Cité, CNRS, Epigenetic Chemical Biology, UMR 3523, F-75724 Paris, France; Université Paris Cité, CNRS, Epigenetics and Cell Fate, F-75013 Paris, France; Université Côte d’Azur, Inserm, CNRS, IRCAN, Nice, France; Université Paris Cité, CNRS, Institut Jacques Monod, F-75013 Paris, France; Institut Pasteur, Université Paris Cité, CNRS, Epigenetic Chemical Biology, UMR 3523, F-75724 Paris, France; Université Côte d’Azur, Inserm, CNRS, IRCAN, Nice, France; Université Paris Cité, CNRS, Epigenetics and Cell Fate, F-75013 Paris, France; Department of Biochemistry, Kyushu University Graduate School of Medical Sciences, Fukuoka, Fukuoka 812-8582, Japan; Université Paris Cité, CNRS, Epigenetics and Cell Fate, F-75013 Paris, France

## Abstract

Epigenetic mechanisms are essential to establish and safeguard cellular identities in mammals. They dynamically regulate the expression of genes, transposable elements and higher-order chromatin structures. Consequently, these chromatin marks are indispensable for mammalian development and alterations often lead to disease, such as cancer. Bivalent promoters are especially important during differentiation and development. Here we used a genetic screen to identify new regulators of a bivalent repressed gene. We identify BEND3 as a regulator of hundreds of bivalent promoters, some of which it represses, and some of which it activates. We show that BEND3 is recruited to a CpG-containg consensus site that is present in multiple copies in many bivalent promoters. Besides having direct effect on the promoters it binds, the loss of BEND3 leads to genome-wide gains of DNA methylation, which are especially marked at regions normally protected by the TET enzymes. DNA hydroxymethylation is reduced in *Bend3* mutant cells, possibly as consequence of altered gene expression leading to diminished alpha-ketoglutarate production, thus lowering TET activity. Our results clarify the direct and indirect roles of an important chromatin regulator, BEND3, and, more broadly, they shed light on the regulation of bivalent promoters.

## Graphical Abstract

**Graphical Abstract:**
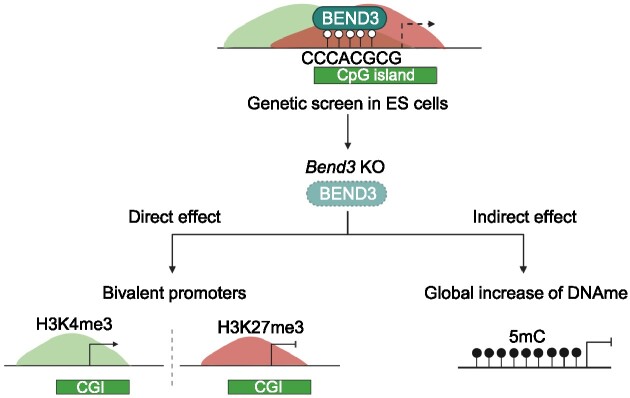

## INTRODUCTION

The development and life of organisms depend on the execution of precise transcriptional programs, which allow the different cell types to respond to stimuli and fulfill their biological function. Part of the information underpinning these programs is hardwired in the genome: promoters and enhancers drive the context-dependent expression of genes, in response to transcriptional regulators that recognize specific sequences (1). In addition to this sequence-based information, chromatin constitutes an additional regulatory layer that is vital for proper gene expression. Nucleosomes are omnipresent in the genome, and the modifications borne by their histone tails profoundly influence gene expression. This regulation occurs through modulating chromatin compaction and directly recruiting or repelling nucleosome remodelers, as well as transcriptional activators and repressors (2). These chromatin marks can be remodeled in response to certain cues and can be (at least partially) carried through mitosis, thereby constituting an epigenetic ‘memory’ (3).

Chromatin marks do not just turn genes ‘On’ or ‘Off’, at least one other state exists, the ‘Poised’ situation. Poised genes simultaneously harbor activating and repressive histone marks on their promoter, rendering it ‘bivalent’ (4). Bivalency is especially prevalent on key developmental genes, where it may facilitate their rapid induction during developmental transitions when they resolve into active or repressed states. Bivalency exists in different cell types, yet a particularly useful model to study bivalency is mouse ES cells, in which poised promoters were in fact initially discovered (5–7). Work with this system over the past 15 years has led to several key insights, yet the molecular foundations are not well understood. One important question that remains to be elucidated is: which factors maintain bivalent promoters in a poised state?

This paper aims to identify regulators of bivalent genes using minimally biased discovery tools (8). To accomplish this, we built a reporter cell line in mouse ES cells and carried out a primary CRISPR KO screen, followed by a custom secondary screen using Fluidigm screening. Through this approach, we discover that BEND3, a transcription factor previously known to act at ribosomal DNA (9), G4 quadruplexes (10) and pericentric heterochromatin (11,12), binds and regulates hundreds of bivalent promoters, via a consensus site that contains a CpG and is only bound when unmethylated. This confirms a recent report, which arrived at similar conclusions by independent approaches (13). Beyond confirming these data, we describe a new role of BEND3, as we show that *Bend3* loss leads to a decrease of DNA hydroxymethylation and a global increase of DNA methylation, which indirectly affect hundreds of non-bivalent genes.

## MATERIALS AND METHODS

### Cell culture

Mouse ES cells were grown on gelatin-coated dishes in serum/LIF medium containing DMEM/GlutaMAX supplemented with 15% fetal bovine serum (FBS), non-essential amino acids (NEAA), penicillin/streptomycin and 1000 U/ml leukemia inhibitory factor (LIF). For certain experiments, ESCs were adapted to 2i/Vitamin C/LIF medium containing serum-free DMEM-F12 and Neurobasal medium supplemented with 1% N2, 2% B27, 100 μg/ml ascorbic acid, 1 μM of PD0325901 and 3 μM of CHIR99021. The cells were incubated in a humidified atmosphere at 37°C under 5% CO_2_.

### Cloning of sgRNA, transfection and transduction in mES cells

Single guide RNAs (sgRNAs) were designed using Benchling/CRISPOR software. The sgRNAs were cloned into either vector PX459 (Addgene #62988) or vector lentiCRISPRv2 (Addgene #52961). For the transfection of ESCs, we used an Amaxa 4D nucleofector (Lonza). Lentiviral particles were produced by calcium phosphate transfection of HEK293T with psPAX2 and pMD2.G plasmids in a BSL3 tissue culture system. Oligonucleotide sequences are listed in Supplementary Table S1.

### Generation of DASH (dazl-mScarlet-Hygromycin^R^) reporter cell line

A P2A-mScarlet-T2A-HygroR reporter cassette (GenScript) was inserted in-frame into exon 6 of the mouse *Dazl* gene. The cassette was flanked by *Dazl* homology arms corresponding to the endogenous intron 5-exon 6 and intron 6 sequences, respectively. To prevent re-cutting by Cas9 after cassette insertion by homologous recombination, the Protospacer Adjacent Motif (PAM) sites of the two sgRNAs targeting *Dazl* exon 6 were mutated within the homology arms. The synthesized cassette was cloned into pUC57-Simple. Two sgRNAs targeting *Dazl* exon 6 were cloned into the pSpCas9(BB)-2A-GFP backbone (Addgene #48138). Homologous incorporation of the reporter cassette into one of the *Dazl* alleles was confirmed by PCR and sequencing. Oligonucleotide sequences are listed in Supplementary Table S1.

### Uhrf1 *KO: validation of DASH mES cells*

Two sgRNAs targeting *Uhrf1* exon 2 were designed, cloned in the PX459 vector and transfected into DASH cells to test the reporter, followed by a short pulse of 1 μg/ml puromycin for 24 h. Knockout efficiency was assessed by western blotting. Oligonucleotide sequences are listed in Supplementary Table S1.

### *CRISPR KO screen and generation of clonal* bend3 *KO lines*

The screen and the CRISPR KOs were performed as detailed in Gupta *et al.* (14). Screen results (top 100 hits) are listed in Supplementary Table S2.

### Microfluidic RT-qPCR (fluidigm): cDNA synthesis, RT-qPCR and analysis

Total RNA was extracted and quantified as described above. RNA quality and integrity were analyzed by capillary electrophoresis with the Fragment Analyzer (Agilent Technologies) to calculate the RNA quality number (RQN) for each sample. Defined on a scale ranging from 1 to 10, the mean RQN of the 52 samples was 9.9, indicating very good RNA quality. The cDNA synthesis was performed using Reverse Transcription Master Mix (Fluidigm) according to the manufacturer's protocol in a final volume of 5 μl containing 200 ng total RNA. Reverse transcription was performed using a Nexus Thermocycler (Eppendorf) with the following parameters: 5 min at 25°C, 30 min at 42°C followed by heat-inactivation of reverse transcriptase for 5 min at 85°C and immediately cooled to 4°C. cDNA samples were diluted 5× by adding 20 μl of low TE buffer (10 mM Tris, 0.1 mM EDTA, pH 8.0) (TEKNOVA) and stored at –20°C before specific target pre-amplification. Diluted cDNA was used for multiplex pre-amplification in a total volume of 5 μl containing 1 μl of 5× PreAmp Master Mix (Fluidigm), 1.25 μl of cDNA, 1.25 μl of pooled assay (Exiqon) with an original concentration of each assay of 10 μM and 1.5 μl of nuclease-free water. The cDNA samples were subjected to pre-amplification with the following conditions: 95°C for 2 min, followed by 10 cycles at 95°C for 15 s and 60°C for 4 min. The pre-amplified cDNA was diluted 5× by adding 20 μl of low TE buffer and stored at –20°C before RT-qPCR. The expression of 48 target genes was quantified in 52 samples by quantitative PCR using the high-throughput platform BioMark HD System on two 48 × 48 GE Dynamic Arrays (Fluidigm). Pre-amplified cDNA was used in a total volume of 15 μl containing 7.5 μl of 2× Gene Expression PCR Master Mix (Thermo Fisher Scientific), 3 μl of pre-amplified cDNA, 0.75 μl of 20× EvaGreen (Biotium), 1.5 μl of each primer (Exiqon) and 2.25 μl nuclease-free water. Thermal conditions for qPCR were: 50°C for 2 min before initial heat-activation of DNA polymerase at 95°C for 10 min, followed by 40 cycles at 95°C for 15 s and 60°C for 1 min. Melt curve analysis was performed with an increase of 0.2°C/s, starting from 65°C for 5 s and ending at 95°C. The parameters of the thermocycler were set with ROX as a passive reference and a single probe EvaGreen as a fluorescent detector. To determine the quantification cycle Cq, data were processed by an automatic threshold for each assay, with linear derivative baseline correction using BioMark Real-Time PCR Analysis Software 4.0.1 (Fluidigm). The quality threshold was set at the default setting of 0.65. Second data analysis was performed using R software to measure the relative gene expression using the comparative Cq method with the efficiency corrected method of Pfaffl, after normalization with reference genes (Cq mean of *Actinb*, *Ppia* and *Rplp0*). Fold change between experimental and control groups was calculated for each sample as the difference of Cq between reference genes and the gene of interest (GOI) in control and experimental conditions.

### Flow cytometry

The number of cells expressing mScarlet was determined in the ImagoSeine Core Facility (Institut Jacques Monod) using the Influx or FACSAria Fusion cell sorter (BD Biosciences) with yellow laser (561 nm). The percentage of positive cells was determined by flow cytometry using the mScarlet signal intensity threshold (minus background fluorescence from wild-type mESCs). Data were analyzed using FlowJo software (BD Biosciences).

### Isolation of genomic DNA

Genomic DNA was isolated from cells by a 200 μg/ml proteinase K treatment at 55°C overnight, followed by 20 μg/ml RNase A treatment at 37°C for 1 h, and extraction using the standard phenol/chloroform/alcohol method. Genomic DNA was suspended in water and quantified with the Qubit dsDNA BR Assay kit on a Qubit 2.0 Fluorometer (Thermo Fisher Scientific) system (Agilent), and only samples with DNA Integrity Number >9 were used for subsequent analysis.

### Identification of CRISPR-induced mutation

Genomic DNA was isolated as described above. sgRNA target region amplicons were generated by PCR using 100 ng genomic DNA, 100 nM primers and Platinum Taq polymerase (Thermo Fisher Scientific). The amplicons were then sequenced. Oligonucleotide sequences are listed in Supplementary Table S1.

### Rescue experiments, piggyBac system

In the rescue experiments, the *Bend3* coding sequence was synthesized (GenScript), with silent mutations incorporated within the PAM to prevent cutting by the sgRNAs that may still be expressed in the KO clone cell lines. This CDS sequence was cloned into a piggyBac vector and co-transfected with PB transposase for stable insertion (15). Empty piggyBac vectors served as controls. Transfected cells were selected with 5 μg/ml of blasticidin for 5 days and processed for phenotypic and molecular assays.

### RT-qPCR

Total RNA was extracted from cells using the RNeasy Plus Mini kit (Qiagen) and quantified using the Qubit RNA BR Assay kit on a Qubit 2.0 Fluorometer (Thermo Fisher Scientific). One microgram of total RNA was reverse transcribed using SuperScript IV Reverse Transcriptase (Thermo Fisher Scientific) and Oligo dT primers (Promega). *Actinb*, *Ppia* and *Rplp0* were used for normalization. Oligonucleotide sequences are listed in Supplementary Table S1.

### Western blotting

Cells were harvested and lysed in RIPA buffer with protease inhibitors (Thermo Fisher Scientific), sonicated in a Bioruptor (Diagenode) with 30s ON/30s OFF for 5 min, then centrifuged at 16 000g for 5 min at 4°C. The supernatant was collected and quantified by BCA assay (Thermo Fisher Scientific). Thirty micrograms of protein extract per sample were mixed with NuPage 4× LDS Sample Buffer and 10× Sample Reducing Agent (Thermo Fisher Scientific) and denatured at 95°C for 5 min. Samples were separated in precast SDS-PAGE 4–12% gradient gels (Thermo Fisher Scientific) at 120 V electrophoresis for 90 min and blotted onto a nitrocellulose membrane (Millipore). The membranes were blocked with 5% fat-free milk/PBS for 1 h at RT and incubated overnight at 4°C with the appropriate primary antibody; after washing three times with PBS/0.1% Tween20, the membranes were incubated with homologous fluorescent secondary antibodies and visualized with the LI-COR Odyssey-Fc imaging system. The following antibodies were used in this study: α-BEND3 (Abcam #ab220896, 1:1000), α-DAZL (Abcam #ab34139, 1:500), α-DNMT3A (Cell Signaling #3598, 1:500), α-DNMT3B (Novus #56514, 1:500), α-UHRF1 (SCBT #98817; 1.1000): 1000), α-V5 (Abcam #ab206566, 1:1000), α-TUBULIN (Abcam #7291, 1:1000) and α-GAPDH (Abcam #ab9485, 1:1000). The following secondary antibodies were used in this study: IRDye 800CW Donkey α-Rabbit (Licor #926-32213, 1:5000) and IRDye 680RD Donkey α-Mouse (Licor #926-68072, 1:5000).

### DNA methylation analysis: luminometry methylation assay (LUMA)

To assess global CpG methylation, 500 ng of genomic DNA was digested in parallel reactions with MspI + EcoRI and HpaII + EcoRI (NEB), with EcoRI as an internal reference. The rate of CpG methylation is defined as the HpaII/MspI ratio. Samples were analyzed using a PyroMark Q24 Advanced Pyrosequencer (Qiagen).

### DNA methylation analysis: LC–MS/MS

Genomic DNA was extracted with RNase A as described above, plus an additional digestion step: 1 μg of DNA was treated with 10U DNA Degradase Plus (ZymoResearch) at 37°C for 4 h, followed by inactivation of the enzyme at 70°C for 20 min and then Amicon Ultra-0.5 ml 10 K centrifugal filters (Merck Millipore) were used to filter the solution. The reaction mixture retained on the centrifuge filter was processed for LC–MS/MS analysis; analysis of total 5-mdC and 5-hmdC concentrations was performed using a Q exactive mass spectrometer (Thermo Fisher Scientific). The instrument was equipped with an electrospray ionization source (H-ESI II Probe) coupled to an Ultimate 3000 RS HPLC (Thermo Fisher Scientific). A ThermoFisher Hypersil Gold aQ chromatography column (100 mm × 2.1 mm, 1.9 μm particle size) heated to 30°C was injected with digested DNA. The flow rate was set to 0.3 ml/min and the column was run for 10 min in isocratic eluent consisting of 1% acetonitrile in water containing 0.1% formic acid. Parent ions were fragmented in positive ion mode, parallel reaction monitoring (PRM) mode at 10% normalized collision energy; MS2 resolution was 17 500, AGC target was 2e5, maximum injection time was 50 ms, and separation window was 1.0 *m*/*z*. The inclusion list contained the following masses: dC (228.1), 5-mdC (242.1) and 5-hmdC (258.1). Extracted ion chromatograms (±5 ppm) of basic fragments were used for detection and quantification (dC: 112.0506 Da; 5-mdC: 126.0662 Da; 5-hmdC: 142.0609). Calibration curves were previously generated using synthetic standards in the ranges of 0.2–10 pmol injected for dC and 0.02 to 10 pmol for 5mdC and 5hmdC. Results were expressed as % of total dC.

### RNA-sequencing: library preparation

A total amount of 1 μg total RNA per sample was used as input material for the RNA sample preparations. RNA samples were spiked with ERCC RNA Spike-In Mix (Thermo Fisher Scientific). Sequencing libraries were generated using NEBNext UltraTM RNA Library Prep Kit for Illumina (NEB) following the manufacturer's recommendations. Briefly, mRNA was purified from total RNA using poly-T oligo-attached magnetic beads. Fragmentation was carried out using divalent cations under elevated temperature in NEBNext First Strand Synthesis Reaction Buffer (5×). First-strand cDNA was synthesized using a random hexamer primer and M-MuLV Reverse Transcriptase (RNase H–). Second strand cDNA synthesis was subsequently performed using DNA Polymerase I and RNase H. In the reaction buffer, dNTPs with dTTP were replaced by dUTP. The remaining overhangs were converted into blunt ends via exonuclease/polymerase activities. After adenylation of 3′ ends of DNA fragments, NEBNext Adaptor with hairpin loop structure was ligated to prepare for hybridization. To select cDNA fragments of preferentially 250–300 bp in length, the library fragments were purified with the AMPure XP system (Beckman Coulter). Then 3 μl USER Enzyme (NEB) was used with size-selected, adaptor-ligated cDNA at 37°C for 15 min followed by 5 min at 95°C before PCR. Then PCR was performed with Phusion High-Fidelity DNA polymerase, Universal PCR primers and Index (X) Primer. Lastly, products were purified (AMPure XP system) and library quality was assessed on the Agilent Bioanalyzer 2100 system.

### RNA-sequencing: read alignment

FASTQ reads were trimmed using Trimmomatic (v0.39) and parameters: ILLUMINACLIP:adapters.fa:2:30:10 SLIDINGWINDOW:4:20 MINLEN:36. Read pairs that survived trimming were aligned to the mouse reference genome (build mm10) using STAR (v2.7.5c) and default single-pass parameters. PCR duplicate read alignments were flagged using Picard-tools (2019) MarkDuplicates (v2.23.4). Uniquely aligned, non-PCR-duplicate reads were kept for downstream analysis using Samtools view (v1.10) and parameters: -q 255 -F 1540. Gene expression values were calculated over the mm10 NCBI RefSeq Genes annotation using VisRseq (v0.9.12) and normalized per million aligned reads per transcript length in kilobases (RPKM). Bigwig files were generated using deeptools bamCoverage (v3.3.0) using counts per million (CPM) normalization and visualized in IGV (v2.8.9).

### RNA-seq: differential expression and PCA plots

DESeq2 (v1.30.0) was employed using apeglm LFC shrinkage to calculate differential expression (Supplementary Table S3). Genes or transposable elements were categorized as significantly differentially expressed if they showed an expression fold-change ≥2 and associated adjusted *P*-value <0.01. PCA plots were generated using variance-stabilized RNA-seq read counts using ‘varianceStabilizingTransformation’, and plotted using the DESeq2 function ‘plotPCA’ and visualized using the R package ‘ggplot2’ (v3.3.3) in R (v4.0).

### RNA-seq: transposable element quantification

RepeatMasker (last updated 2012-02-06) was downloaded from the UCSC Table Browser. To measure the expression of transposable element families, PCR duplicates were removed and all reads, including uniquely mapped and multi-mapped reads, were enumerated using VisRseq. Multi-mapped reads were counted once, and all individual elements were aggregated to calculate family-wide expression in read count (for differential expression analysis) and RPKM values.

### Chromatin immunoprecipitation (ChIP-seq)

Ten million cells were cross-linked (1% formaldehyde, 5 min RT); the cross-linking stopped by adding glycine at 125 mM final). After a PBS wash the cells were resuspended in swelling buffer (0.5% NP-40, 0.85 mM KCl, 1 mM PMSF, 5 mM PIPES pH 8.0; protease inhibitors) and incubated on ice for 20 min. After centrifugation, the cell nuclei were resuspended in IP Buffer (0.1% SDS, 1% Triton X-100, 3 mM EDTA, 1 mM PMSF, 150 mM NaCl, 25 mM Tris–HCl pH 8.0; with protease inhibitors) and sonicated in a Bioruptor Pico (Diagenode) for 5 min (30 s ON/30 s OFF continuously) to generate 200–500 bp fragments. The fragmented chromatin (50 μg) was immunoprecipitated overnight at 4°C in IP buffer with 1 μg of antibody. The following antibodies were used in this study: α-BEND3 (Abcam #ab220896), α-V5 (Abcam #ab206566), IgG (Abcam #ab46540). Subsequent steps were performed using the Pierce Magnetic ChIP Kit (Thermo Fisher Scientific). The resulting DNA was purified using the ChIP DNA Clean&Concentrator Kit (Zymo Research), and libraries were prepared using the KAPA HyperPrep Kit (Roche) according to the manufacturer's instructions.

### ChIP-seq analysis

FASTQ reads were trimmed with Trimomatic (v0.39), parameters ILLUMINACLIP:ILLUMINA_adapters.fa:2:30:10 SLIDINGWINDOW:4:20 MINLEN:36 Alignment of trimmed reads was performed in –local mode in Bowtie2 (v2.4.1). After alignment, duplicate-marked bam files were created using Picard (v2.23.4) CleanSam, SamFormatConverter, SortSam and Markduplicates. The resulting bam files were then converted to bigwig with deptools (v3.3.0) Bamcoverage and the option –ignoreDuplicates -normalizeUsing CPM -minMappingQuality 10 -ignoreForNormalization chrX chrY chrM. Peaks were called using MACS2 with default settings and motif enrichment analysis was carried out in HOMER. For datasets that were already publicly available, SRA files were downloaded from NCBI GEO using the Eearch and efetch utilities (v15.9) and converted to FASTQ using SRAtoolkit (v2.8.0). Published datasets were reanalyzed from GSE98149 (16).

### Whole-genome-bisulfite sequencing (WGBS)

Genomic DNA was extracted as described for LC–MS/MS; library preparation for WGBS was performed using the previously described tPBAT protocol (17,18). For library preparation, 100 ng of genomic DNA spiked with 1% (w/w) unmethylated lambda DNA (Promega) was used. Sequencing was performed by MacroGen Japan, Inc. using a HiSeq X Ten system; 8 lanes were allocated for analysis of 20 samples. Sequenced reads were mapped in BMap and compiled in an in-house pipeline as previously described, and custom scripts were archived using GitHub (https://github.com/FumihitoMiura/Project-2). Basic metrics for the methylome data are described in Supplementary Table S4. DNA methylation levels on CpGs covered by at least five reads were averaged over the target region of 2 kb bins genome-wide, over CpG island (cpgIslandExt, *n* = 16 023, last updated 2012-02-09), enhancer elements (Ensembl Regulatory features release 81, *n* = 73 796), promoters (NCBI Refseq, TSS ± 1 kb, *n* = 24 371), gene bodies and transposable elements (RepeatMaster, *n* = 5 147 736).

## RESULTS

### Principle of the genetic screen

**Figure 1.**
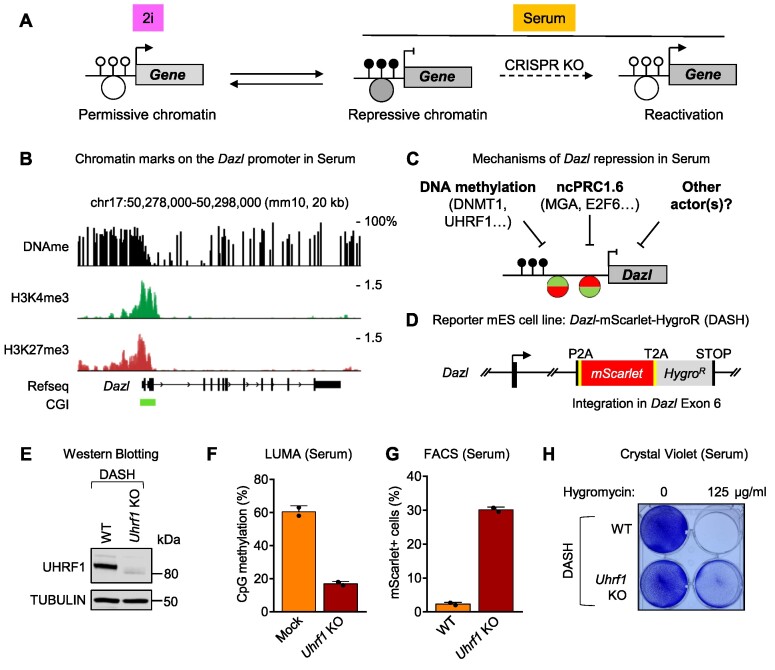
Principle of the reactivation screen, generation and validation of the DASH reporter cell line. (**A**) Mouse ES cells can be grown in 2i medium or in serum. A number of genes acquire DNA methylation (black lollipops) and repressive histone marks (large gray circle) in serum, which inhibits their expression. Performing a CRISPR loss-of-function screen in serum should lead to the identification of repressors. (**B**) The *Dazl* gene promoter contains a methylated CpG island, the activating histone mark H3K4me3 and the repressive histone mark H3K27me3: it is bivalent. (**C**) DNA methylation and the ncPRC1.6 complex are already known to repress *Dazl* expression in serum. We searched for new repressors. (**D**) We built the DASH reporter cell line, in which two positively selectable markers (mScarlet and HygroR) are inserted into exon 6 of *Dazl*. The other allele of *Dazl* in DASH cells is WT. (**E**) CRISPR KO of *Uhrf1* in DASH cells is confirmed by western blotting. (**F**) LUMA confirms that *Uhrf1* KO in DASH cells leads to a global loss of DNA methylation. (**G**) *Uhrf1* KO in DASH cells results in mScarlet reactivation. (**H**) *Uhrf1* KO in DASH cells results in Hygromycin resistance; surviving cells stained with crystal violet.

Epigenetic silencing is dynamic in mouse ES cells and responds to culture conditions. In particular, a number of genes are expressed in 2i condition, but are repressed when the cells are switched to serum (Figure 1A). The principle of our screen was to select one such gene and replace it with selectable markers to carry out a CRISPR KO screen in serum, and to positively select the cells in which the KO caused a loss of silencing (Figure 1A). To select the gene in question, we considered three criteria: mechanism(s) of epigenetic repression, biological function(s) of the gene and expression level in repressed and induced condition (to ensure high enough expression of the selection markers). These criteria led us to select *Dazl* as our target of choice. *Dazl* encodes an RNA-binding protein which is critical during gametogenesis (19), but it is also expressed during other developmental events, as in the 2-cell-embryo (20). The promoter of *Dazl* overlaps a large CpG island (1.1 kb); in serum-grown ES cells, the 5′ half of the CpG island is heavily methylated on CpGs, while the 3′ half is mostly void of DNA methylation. In addition, the promoter of *Dazl* contains both the activating H3K4me3 and the repressive H3K27me3 marks and can therefore be classified as a bivalent promoter (Figures 1B and S1A). *Dazl* expression is low in serum, however genetic experiments have shown that this repression can be lifted either by impeding DNA methylation (21,22), or by interfering with the ncPRC1.6 complex (23,24). One of our goals was to identify new actors in this process (Figure 1C).

Having chosen *Dazl* as our reporter gene of interest, we then used CRISPR/Cas9-guided homologous recombination to insert two selectable markers in the gene: mScarlet, encoding a bright red fluorescent protein (25), and Hygro^R^, a bacterial enzyme that renders cells resistant to Hygromycin. The two markers were inserted in-frame in the *Dazl* exon 6 and were separated by self-cleaving peptides (Supplementary Figure S1B). Recombinant clones were picked and characterized by genomic PCR and sequencing. One heterozygous integrant was further characterized; it contains one wild-type *Dazl* allele, and the mScarlet-Hygro^R^ insertion in the other allele (Figure 1D). We will refer to this line as the DASH (*Dazl*-Scarlet-Hygro) reporter line.

We next carried out a proof-of-concept experiment to verify that the selectable markers could be induced in serum upon the KO of an epigenetic repressor. For this, we used CRISPR to knock out a key factor in DNA methylation maintenance, UHRF1 (Supplementary Figures S1C and S1E). As expected (26,27), the *Uhrf1* KO caused a global loss of DNA methylation as seen by LUMA (Figure 1F). The DASH cells grown in serum became mScarlet-positive and Hygromycin-resistant upon *Uhrf1* KO (Figures 1G, H, and S1D), confirming the feasibility of a genetic screen.

### Primary screen

**Figure 2.**
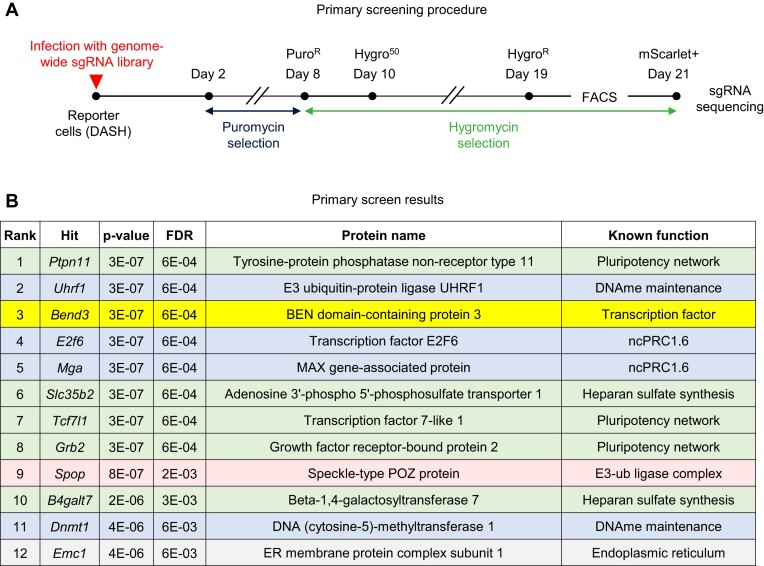
Primary screening approach and results. (**A**) Timeline of the genetic screen. (**B**) Results of the MAGeCK analysis. The top 12 hits are shown, color-coded according to class. The hits in blue were already known and were expected in the list. The hits in green influence cellular pluripotency. Pink, red and yellow: other hits.

We grew the DASH cells in serum and infected them with a lentiviral library of ∼80 000 vectors co-expressing Cas9 and sgRNAs targeting ∼20 000 genes (28). The multiplicity of infection was ∼0.1, the coverage ∼150 infected cells per guide and two independent screens were carried out in parallel. After an initial Puromycin selection to eliminate non-infected cells, Hygromycin selection was applied and the fluorescent cells were purified by FACS (Figure 2A). The sgRNAs present in these cells were amplified by PCR and sequenced by NGS, and the sequencing data statistically analyzed using MAGeCK (29). This procedure yielded an ordered list of candidates, ranked by *P*-value, and a highly stringent cutoff (*P*-value lower than 5 × 10^−6^ and FDR lower than 0.01) yielded a list of 12 hits (Figure 2B). Four of the top 12 hits (ranked #2, 4, 5 and 11 and shown in light blue in Figure 2B) correspond to factors already known to repress *Dazl*: UHRF1, E2F6, MGA and DNMT1. Their presence on the list was expected and supports the validity of the screen. Five of the hits (in light green in Figure 2B) correspond to genes involved in pluripotency or in Heparan Sulfate synthesis.

### Secondary screen on the top hits and focus on BEND3

We next sought to validate the results of the primary screen while also prioritizing the hits for further analysis. For this, we carried out a secondary screen based on gene expression analysis.

We first generated, by lentiviral infection with CRISPR/Cas9 vectors, new KO populations for ten of the 12 top hits (and 3 different negative control populations), as depicted in Supplementary Figure S2A. For technical reasons, *Grb2* was excluded from the analysis and, instead of knocking out *B4galt7*, we targeted *B3galt6*, a gene in the same complex and with the same function. Rather than carrying out a whole-genome RNA-seq on each hit, we examined the expression of 21 genes of interest (along with 3 normalizers) in the aforementioned knockouts using the Fluidigm technology.

The genes are color coded in Supplementary Figure S2B and belong to different functional classes: besides the mScarlet reporter and *Dazl* itself, there were other germline genes (*Asz1, Hormad1, Fkbp6* and *1700013h16Rik*); markers of naive ES cells (*Nanog, Prdm14, Zfp42/Rex1*); repeated elements (*MERVL, MMERVK10C, IAPEz, IAP, MLV, MusD, LINE1* and *GLN*); and finally epigenetic regulators (*Uhrf1, Dnmt1, Dnmt3a, Dnmt3b, Dnmt3l, Tet1* and *Tet2*).

Unsupervised analysis of the Fluidigm data revealed that the KOs were segregated into 4 clusters. Cluster number 1 contained both the *Dnmt1* and *Uhrf1* KOs, which expectedly had very similar expression profiles. In accordance with published data (30), these two KOs reactivated the expression of all germline genes, as well as certain repeats, in particular GLN. The *Ptpn11* KO was the third and final member of this cluster.

Cluster number 2 included *Slc35b2* and *B3galt6* KOs, along with *Mga* and *E2f6*, which behaved similarly, also as predicted. Unsurprisingly (23,24), all germline genes were reactivated in this cluster.

The third cluster contained the *Bend3*, *Spop* and *Tcf7l1* KOs. Contrary to cluster #2, the germline genes were either unaffected or only slightly upregulated in this cluster. The fourth cluster contained all three negative controls, along with the *Emc1* KO. *Dazl* was not reactivated in this cluster, and the other genes were similarly unaffected.

This analysis leads to several useful conclusions. First, 10 out of 11 new KO populations (91%) displayed the induction of *Dazl* that was also observed in the primary screen, therefore validating the hits. The only false positive was *Emc1*. Secondly, hits affecting DNA methylation or ncPRC1.6 form separate clusters. Third, three hits generate a gene expression pattern that resembles neither DNA methylation mutants nor the ncPRC1.6 mutants. These potentially novel hits are *Bend3*, *Spop* and *Tcf7l1*. Of those three hits, *Spop* encodes an adaptor protein for an E3 ubiquitin ligase complex (31,32) and as it seemed least likely to act directly at the *Dazl* promoter we did not pursue it further in this study. *Tcf7l1* is a transcription factor that inhibits the naive state in mouse ES cells (33); as a consequence, cells lacking TCF7L1 tend to remain in a 2i-like state, even when grown in serum (34). Because cells in 2i express *Dazl*, it seemed possible that the upregulated expression of *Dazl* in the *Tcf7l1* KO cells was merely an indirect reflection of a change of cellular identity towards a more naive state. The third hit within the cluster was *Bend3*, which encodes a chromatin protein (11) known to repress transcription at the rDNA (9). As BEND3 had suitable characteristics for being a new direct repressor of *Dazl*, we decided to investigate it further.

### *Isolation of* bend3 *mutant clones and rescue experiments*

**Figure 3.**
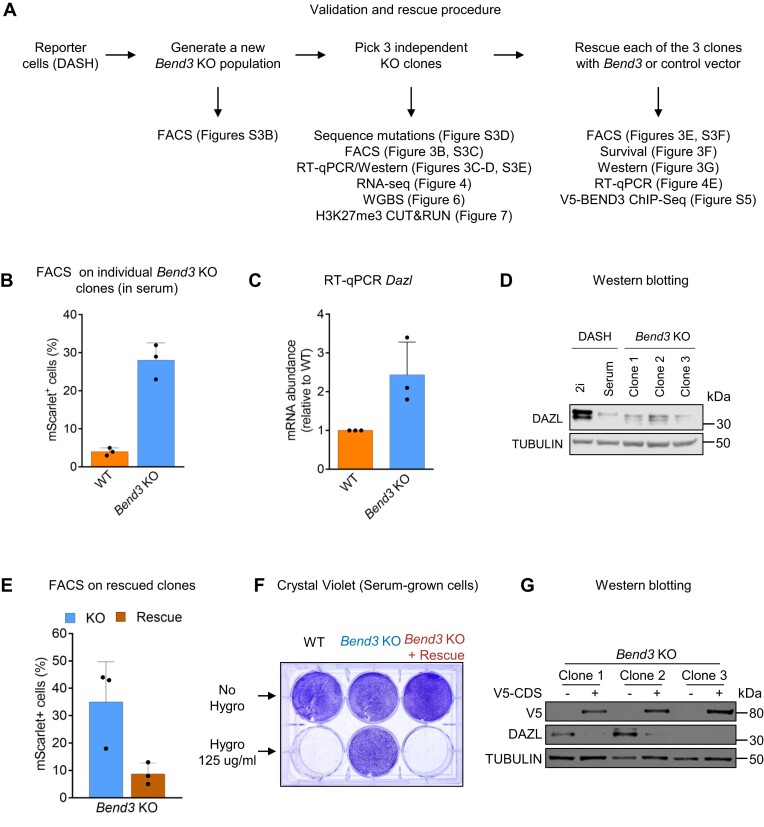
BEND3 represses Dazl expression. (**A**) Procedure for validation of *Bend3* as a *bona fide* hit. (**B**) FACS analysis on serum-grown cells. (**C**) RT-qPCR on serum-grown cells. (**D**) Western blotting on serum-grown cells. (**E**) FACS analysis on serum-grown *Bend3* mutant cells, rescued with empty vector or with a *Bend3*-expressing vector. (**F**) Survival assay on rescued cells. Only one of the three clones is shown, for clarity. (**G**) Western blotting shows expression of the V5-tagged BEND3 protein in rescue cells, and concomitant repression of DAZL protein expression. The three independent clones are shown.

To validate the results of the CRISPR screen, we generated new *Bend3* mutant populations (Figure 3A). For this, we infected DASH cells with a mix of two distinct lentiviruses, each expressing Cas9 and a specific sgRNA targeting *Bend3* (Supplementary Figure S3A). As *Bend3* produces two protein isoforms, we targeted the last exon of *Bend3*, which is included in both isoforms.

Two independent *Bend3*-mutant populations showed a roughly 10-fold increase of mScarlet-expressing cells relative to control, similar to the magnitude observed for *Uhrf1* mutant cells (Supplementary Figure S3B). To carry out more precise experiments, we isolated individual *Bend3* mutant clones from these populations (see scheme in Figure 3A). Three clones were isolated, each of them containing a high proportion of mScarlet-expressing cells (25–35%, relative to ∼3% in control cells, Figures 3B and S3C). These clones also expressed more *Dazl* mRNA (Figure 3C) and DAZL protein (Figure 3D) from the non-targeted allele. Upon sequencing their genomic DNA, we found that two clones had non-sense or frameshift mutations at AA 308, removing about two thirds of the protein. The last clone had a small homozygous in-frame mutation removing four amino acids in the first BEN domain (Supplementary Figure S3D). The BEND3 protein was not detectable in the KO clones (Supplementary Figure S3E), suggesting that the mutant forms are unstable.

We next verified that the phenotypes could be rescued by reintroducing a WT cDNA (see scheme in Figure 3A); for this, we used a PiggyBac transposon-based system (30) driving constitutive expression of V5-tagged BEND3. Expression of the functional *Bend3* cDNA in the *Bend3* KO cells decreased the number of mScarlet-positive cells to near-background levels (Figures 3E and S3F), rendered the cells sensitive to Hygromycin (Figure 3F), and silenced the expression of the DAZL protein (Figure 3G).

Together these experiments support the conclusion that BEND3 is a *bona fide* repressor of *Dazl* expression.

### *Transcriptome analysis in* bend3 *mutant cells reveals up- and down-regulated genes*

**Figure 4.**
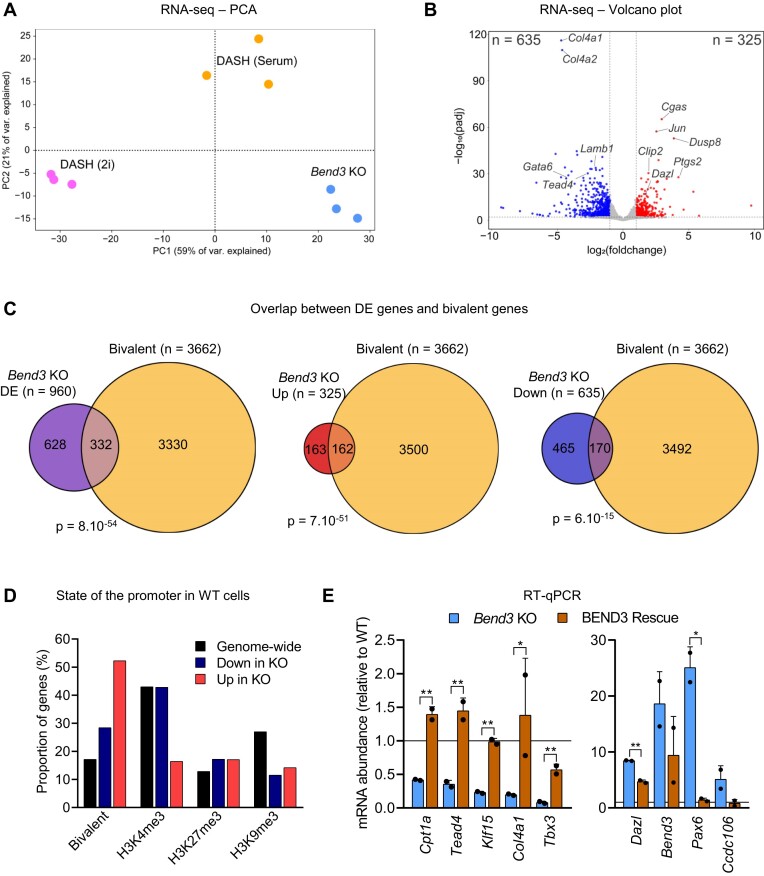
BEND3 regulates the transcription of bivalent promoters. (**A**) Principal Component Analysis on RNA-seq data from the indicated cells (three biological replicates for each condition). (**B**) Volcano plot showing genes that are downregulated (blue) or upregulated (red) in the *Bend3* KO cells. (**C**) Venn diagrams showing the overlap between genes that are differentially expressed (DE, either up or down-regulated), upregulated, or downregulated, between *Bend3* KO and WT, and genes that have a bivalent promoter (35). *P*-value: hypergeometric tests. (**D**) Proportion of genes with the indicated promoter marks within up- or down-regulated genes. (**E**) RT-qPCR following reintroduction of V5-BEND3 in *Bend3* KO cells. Genes that were downregulated upon KO (left panel) regain expression upon rescue, while genes that were upregulated in the KO become repressed upon rescue (right panel). *P*-value: Student's *t*-test, corrected by two-stage Benjamini, Krieger, & Yekutieli FDR procedure. *P < 0.05; **P < 0.01.

To examine more globally the effects of *Bend3* deletion, we performed RNA-seq on the 3 independent mutant clones, grown in serum, which we compared to WT cells (grown in serum or 2i). As shown in Figure 4A, the mutant cells clustered away from the WT cells, and they were highly distinct from 2i grown cells. This supports the idea that the *Bend3* KOs do not reactivate *Dazl* because they enter a 2i-like state.

More genes were repressed than induced in the *Bend3* KO cells (635 versus 325 respectively, Figure 4B and S4A). As expected, *Dazl* was among the induced genes (Figure 4B). A recent publication independently examined the transcriptome of *Bend3*-/- ES cells (31); we compared our respective RNA-seq results and found that the overlap was significant yet small, with only ∼10–20% upregulated or downregulated genes in common (Supplementary Figure S4B). We speculate this dissimilarity results from different procedures for the mutant cell derivation, combined with different cell culture conditions.

We also examined the behavior of transposable elements in the RNA-seq data (Supplementary Figure S4C). The mutation of *Bend3* (left panel) had very little effect on the expression of mouse endogenous retroviruses (ERVs). As a control (right panel), we compared WT cells grown in 2i and serum and predictably found a large induction of ERVs such as IAPs, which validates our analysis pipelines.

Returning to protein-coding genes, we manually inspected some of the differentially expressed (DE) genes and noted that many of them had bivalent marks on their promoter, in line with the recent results of Zhang *et al.* (13). To determine if this observation could be generalized, we used a list of very stringently curated bivalent genes (35). We cross-referenced this list with our RNA-seq data and found that ∼35% of differentially genes in the *Bend3* KO relative to WT are bivalent; this is a highly significant enrichment (*P* = 8 × 10^−54^, Figure 4C). The enrichment is stronger for genes that are upregulated in the *Bend3* mutants (50% of those are bivalent) than for genes that are downregulated in the *Bend3* mutant (27% being bivalent, Figure 4C). Correspondingly, genes induced in the *Bend3* mutant were more likely to have a bivalent than a repressed status in the WT cells (Figure 4D). Bivalent chromatin marks often mark developmental genes in mouse ES cells, and in line with this we found that the genes differentially expressed in *Bend3* KO cells were markedly enriched for developmental regulators (Supplementary Figure S4D).

**Figure 5.**
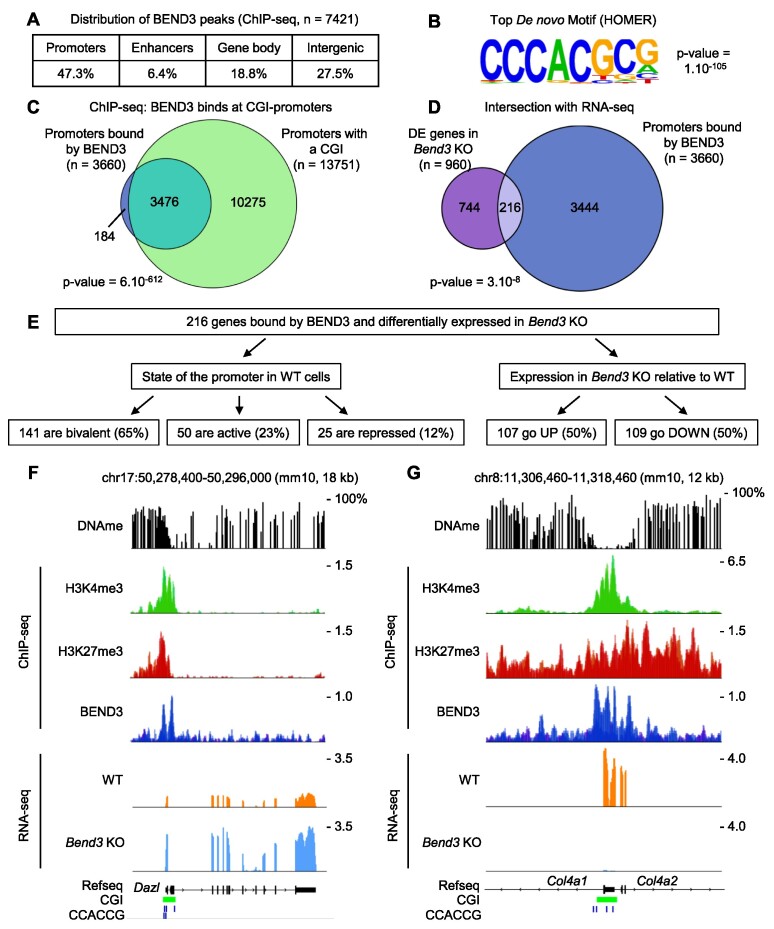
BEND3 binds bivalent promoters via a CpG-containing consensus site. (**A**) Number and distribution of BEND3 peaks detected by ChIP-seq. (**B**) Top motif identified in a de novo motif search on all BEND3 peaks. (**C**) Almost all promoters bound by BEND3 contain a CpG island. *P*-value: hypergeometric tests. (**D**) Intersection between promoters bound by BEND3, and genes Differentially Expressed (DE) in the *Bend3* KO cells. (**E**) Characteristics of the genes directly bound and regulated by BEND3. (**F**) *Dazl* is directly bound and repressed by BEND3. (**G**) Illustration of two bivalent genes directly bound and activated by BEND3: *Col4a1* and *Col4a2*.

Lastly, we performed RT-qPCR in *Bend3* KO cells that had been rescued with the V5-BEND3 construct (Figure 4E). Genes that had been downregulated in the KO cells (*Cpt1a*, *Tead4*, *Klf15*, *Col4a1*, *Tbx3*) were upregulated in the rescue cells; conversely, genes that had been induced in the KO cells (*Dazl*, *Bend3* (measured with primers that do not amplify the rescue construct), *Pax6*, *Ccdc106*) were repressed in the rescue cells (Figure 4E). All of the genes examined in this experiment are directly bound by BEND3, as determined by the ChIP-seq that will be presented later (Figure 5).

Altogether, these data show that removing BEND3 from serum-grown ES cells alters the expression of hundreds of genes, with a high proportion of bivalent genes. Nevertheless, of the ∼3600 bivalent genes, only about 10% are regulated by BEND3 (Figure 4C). To understand this situation, we next sought to identify the direct targets of BEND3.

### ChIP-sequencing identifies the direct targets of BEND3

We then used ChIP-seq to identify genes directly regulated by BEND3. Using an antibody directed against endogenous BEND3, we identified ∼7400 binding peaks in the ES cell genome (Figure 5A). This dataset is highly similar to the dataset of Zhang *et al.* (13), who used the same antibody as we did (Supplementary Figure S5A). We also performed ChIP-seq with an antibody against the V5 tag, in *Bend3* KO cells expressing V5-BEND3. This data obtained overlapped extremely well with the ChIP-seq data obtained on the endogenous protein (Supplementary Figure S5B-C). These two lines of evidence strongly support the quality of our ChIP-seq dataset.

Of the ∼7400 peaks detected in the BEND3 ChIP, 47% occur in promoters, 6% in enhancers, 19% in gene bodies and 28% in other intergenic regions (Figure 5A). A de novo search for motifs enriched in the peaks revealed a highly enriched motif with a strong selection for the core sequence CCCACG (Figure 5B). This motif occurred in 70% of all BEND3 peaks, often in multiple instances (Supplementary Figure S5D), hinting that BEND3 binds it directly, which is supported by recent *in vitro* work (13,36). The overwhelming majority (94%) of promoters bound by BEND3 contain a CpG island, which is a significant enrichment (Figure 5C). BEND3 binding is prevalent at bivalent promoters; in contrast it is extremely rare at promoters bearing H3K9me3 (Supplementary Figure S5E).

We next intersected our RNA-seq data with our BEND3 binding data (Figure 5D). Only a minority of promoters (5.5%) bound by BEND3 become significantly activated or repressed upon KO. Conversely, only about 23% of genes deregulated upon *Bend3* deletion have BEND3 binding at their promoter. The genes bound by BEND3 and differentially expressed in the KO, which are likely to be direct targets, are split equally between upregulated genes and downregulated genes (Figure 5E). Such genes include *Dazl*, which is repressed by BEND3 (Figure 5F), but also *Col4a1* and *Col4a2*, which are activated by BEND3 (Figure 5G). Again, most direct targets of BEND3 (65%) have a bivalent promoter in WT cells (Figure 5E, Supplementary Figure S5E-F); they have a variable number of BEND3 consensus motifs, with 10% having no motif, to 20% having four motifs or more (Figure S5G).

### *Global DNA hypermethylation and reduced 5-hmC in* bend3 *mutant cells*

The results above establish that many of the genes that are up- or down-regulated when BEND3 is absent are not direct targets of the protein. To investigate what might underpin these indirect effects of BEND3, we examined DNA methylation.

**Figure 6.**
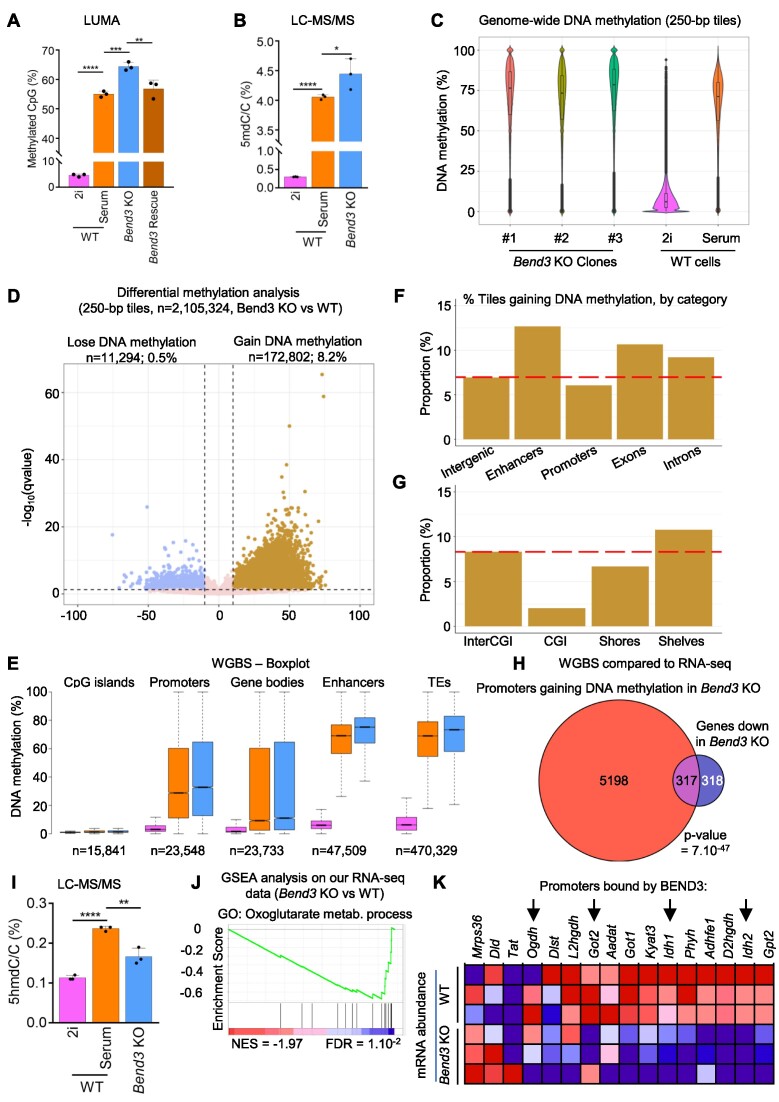
Cells lacking BEND3 have increased DNA methylation and decreased DNA hydroxymethylation. (**A**) Evaluation by a restriction enzyme-based method (LUMA) of the global level of DNA methylation in the indicated cellular contexts. *Bend3* KO cells are hypermethylated, and the expression of a BEND3 rescue construct brings DNA methylation back to WT levels. *P*-value: ANOVA followed by Tukey's post-hoc tests. **P< 0.01; ***P< 0.001; ****P< 0.0001. (**B**) Quantitation of 5-mC abundance in the cells by LC–MS/MS. *P*-value: ANOVA followed by Dunnett's post-hoc tests. *P< 0.05; ****P< 0.0001. (**C**) Distribution of methylation levels on 250-bp tiles containing 5 or more CpGs (*n* = 2.1 million). The three individual *Bend3* KO clones are shown on the left, together with WT cells. (**D**) Differential methylation analysis on 250-bp tiles containing five or more CpGs (*n* = 2.1 million). (**E**) DNA methylation values for the indicated genomic elements in WT cells grown in 2i (pink) or serum (orange), and *Bend3* KO cells grown in serum (blue) (**F**, **G**) Percentage of statistically significant hypermethylated tiles in the indicated genomic compartments, when comparing *Bend3* KO to WT cells. The red dashed line shows the value in intergenic regions, for comparison. See Methods for details. (**H**) Venn diagram showing the overlap between genes that are downregulated in *Bend3* KO and genes whose promoter gains DNA methylation in *Bend3* KO and compared to WT. *P*-value: hypergeometric tests. (**I**) Quantitation of 5-hmC abundance in the cells by LC–MS/MS. p-value: ANOVA followed by Dunnett's post-hoc tests. **P< 0.01; ****P< 0.0001. (**J**) GSEA analysis on the RNA-seq data (Bend3 KO vs. WT). (**K**) RNA-seq data for the genes of ‘Oxoglutarate metabolic process’ GO term. Red: higher expression, blue: lower expression. The genes with promoters directly bound by BEND3 are indicated with arrows.

We first measured global DNA methylation with a method based on restriction enzymes, LUMA (37). The WT cells grown in serum and 2i media served as controls and, as expected, global DNA methylation was low in 2i and high in serum (Figure 6A). Additionally, the level of DNA methylation was higher in *Bend3* mutant cells than in WT cells (both grown in serum). Importantly, rescuing the loss-of-function mutations with a *Bend3*-expressing construct brought the level of DNA methylation back to WT levels (Figure 6A).

To validate these observations with an orthogonal approach, we turned to LC–MS/MS: this experiment confirmed that 5mC was more abundant in the *Bend3* KO cells (Figure 6B).

We then performed Whole-Genome Bisulfite Sequencing (WGBS) on WT cells, and on each individual *Bend3* mutant clone (Supplementary Figure S6A). As anticipated, the 2i-grown WT cells were vastly undermethylated relative to serum-grown WT cells (Figure 6C), and the methylation of genomic tiles in serum-grown WT cells displayed a typical bimodal profile: a minority of tiles unmethylated and most tiles methylated, with a peak around 80% methylation (Figure 6C), lending confidence to our WGBS experiment and analysis. Upon comparison to WT cells, we saw that the *Bend3* mutants had a methylation distribution that was still bimodal but in which the methylated compartment was shifted towards higher methylation levels (Figures 6C and S6B). As the three clones behaved similarly, we aggregated their WGBS reads in subsequent analyses.

To carry out a more precise statistical analysis, we narrowed down our calculations to 250-bp tiles containing 5 or more CpGs, for a total of 2.1 million tiles. Of these, 91.3% do not significantly gain or lose methylation upon *Bend3* mutation, 8.2% gain DNA methylation and 0.5% lose DNA methylation (Figures 6D, S6C, D).

We then investigated the genomic distribution of the DNA methylation gains. The increase was global and affected promoters, enhancers, gene bodies and transposable elements (Figure 6E). Over the whole genome, 8.2% of the tiles gain DNA methylation, as stated above, however the level of increase differed between genomic elements: enhancers had a marked tendency to gain more DNA methylation, while promoters were slightly under average (Figure 6F). Looking at CpG island, we saw that the CpG islands themselves were rarely hypermethylated, the CpG shores gained slightly less methylation, whereas the CpG shelves gained more methylation than average (Figure 6G). In other words, the gains of DNA methylation occur at a distance from the CpG islands, which is exemplified by the gene *Tead4* (Supplementary Figure S6E).

We then crossed our WGBS data with ChIP-seq data reflecting the chromatin state in WT cells (Supplementary Figure S6F). We find that regions that were bivalent or marked by H3K4me3 gain more methylation than average. Regions marked by H3K27Ac, which is typical of enhancers, are also much more likely to gain DNA methylation than the genome average, again suggesting that enhancers are targets for DNA hypermethylation in *Bend3* KO cells.

Next, we intersected our WGBS and RNA-seq data. We find that half of transcriptionally downregulated genes in *Bend3* KO cells gain DNA methylation in their promoter (Figure 6H). The BEND3-bound peaks themselves, or the genes upregulated in *Bend3* KO, gain methylation less than the rest of the genome (Supplementary Figure S6G).

We investigated mechanisms that could possibly underpin the elevated DNA methylation seen in *Bend3* mutant cells. Neither our RNA-seq data (Supplementary Figure S6H), nor our western blotting experiments (Supplementary Figure S6I) suggested that BEND3 removal upregulated *Uhrf1*, *Dnmt1*, *Dnmt3a*, or *Dnmt3b*, which are key actors of DNA methylation. Therefore we speculated that DNA hydroxymethylation might be modified in the *Bend3* KOs. To test this idea, we again used LC–MS/MS, and we indeed saw a significant decrease of 5-hmC levels in *Bend3* mutant cells (Figure 6I). This effect is not due to transcriptional downregulation of the TET enzymes in the absence of BEND3 (Supplementary Figure S6H), so we considered additional mechanisms that could potentially lead to lower TET activity in *Bend3* mutant cells. The TET enzymes are critically dependent on alpha-ketoglutarate, so we tested whether the transcriptional defects occurring in *Bend3* KO cells might impinge on the pathways producing this metabolite. A GSEA analysis of our RNA-seq data showed that there was indeed a significant downregulation of the group of genes involved in alpha-ketoglutarate production (Figure 6J). To investigate this finding in more depth, we examined the expression of individual genes belonging to this GO term group, and intersected the data with our BEND3 ChIP-seq results (Figure 6K). We find that several genes of the group are bound by BEND3 and downregulated in its absence. In particular, *Idh1* and *Idh2*, encoding Isocitrate Dehydrogenase, which produces alpha-ketoglutarate, are both directly activated by BEND3. We hypothesize that the lower *Idh1/Idh2* expression causes lower alpha-ketoglutarate availability, reducing TET activity, and increasing DNA methylation in cells lacking BEND3.

### Integrating the local and global effects of BEND3 on chromatin and transcription

Lastly, we set out to integrate the changes occurring at the chromatin (DNA methylation and histone marks) and transcriptional levels in the *Bend3* mutant cells.

**Figure 7.**
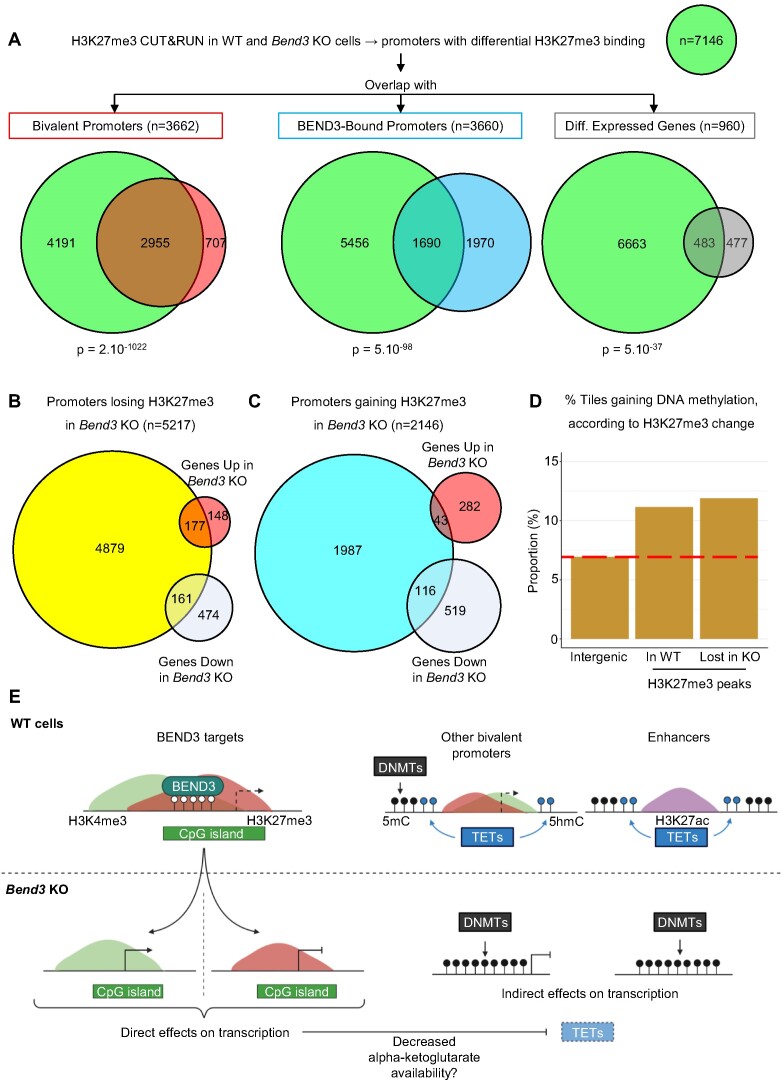
Integrating the direct and indirect roles of BEND3 on chromatin and transcription. (**A**) Summary of the H3K27me3 CUT&RUN results in WT and *Bend3* KO. *P*-value: hypergeometric tests. (**B**) Comparison of H3K27me3 loss and differential gene expression in *Bend3* KO cells. (**C**) Comparison of H3K27me3 gain and differential gene expression in *Bend3* KO cells. (**D**) Percentage of statistically significant hypermethylated tiles in the indicated genomic compartments, when comparing *Bend3* KO to WT cells. The red dashed line shows the value in intergenic regions, for comparison. (**E**) Model for the direct and indirect effects of BEND3 on chromatin and gene expression.

For this, we mapped the key histone modification H3K27me3 by CUT&RUN in ES cells with or without BEND3 (Figure 7A). Bivalent promoters were extremely likely to experience changes in H3K27me3 abundance upon BEND3 removal (Figure 7A, left panel), reinforcing the idea that BEND3 has a key role in their homeostasis. Of the promoters directly bound by BEND3, 46% experienced changes in H3K27me3 occupancy, again a significant enrichment (Figure 7A, middle panel). Finally, of the 960 genes differentially expressed upon BEND3 loss, more than half experienced changes in H3K27me3 presence on their promoter (Figure 7A, right panel). The correlations between BEND3 binding, H3K27me3 presence, changes in gene expression and changes in DNA methylation, are explored statistically in Supplementary Figure S7A, and the examples of *Dazl* and *Col4a1/a2* are shown in Supplementary Figure S7B-C.

We also examined some of the interactions more precisely. For instance, we listed the promoters that lose H3K27me3 when BEND3 is absent (Figure 7B). Fifty-four percent of the upregulated genes were in that category, versus only 25% of the downregulated genes. Therefore, transcriptional induction upon *Bend3* mutation often involves H3K27me3 loss, in line with many of the target genes having bivalent promoters. The opposite was true for promoters that gain H3K27me3 upon BEND3 loss: they contain more downregulated than upregulated genes (Figure 7C).

Lastly, we crossed our CUT&RUN and WGBS data (Figure 7D). The regions that were occupied by H3K27me3 in WT cells were significantly more likely to gain DNA methylation than the genome average. Similarly, the regions that have lost H327me3 in the *Bend3 KO* cells were significantly more hypermethylated (Figure 7D). Further examples are given in Supplementary Figure S7D-E.

Altogether these data lead us to propose an integrated model for the local and global effects on BEND3 on chromatin and gene expression (Figure 7E).

## DISCUSSION

In this work, we set out to identify new regulators of *Dazl* using a CRISPR screen in mouse ES cells. Our primary screen identified a shortlist of 12 candidates, of which 4 were known regulators of *Dazl*. A secondary screen then led us to focus on one particular hit, BEND3. We combined ES cell genetics with various genomic approaches to show that BEND3 regulates not only *Dazl*, but hundreds of bivalent promoters.

A recent article by Rui-Ming Xu, Bing Zhu and their coworkers, also identified BEND3 as a key regulator of bivalent genes (13). Zhang *et al.* reached these conclusions by a wholly different approach, which was the generation of *Bend3-/*- mice and the derivation of ES cells from these mice. We note that, although our conclusions are parallel, some of the specifics are different: for instance, only about 10% of the differentially expressed genes in *Bend3* mutant cells relative to WT are the same between our study and Zhang *et al.* Technical reasons (including genetic background and culture conditions) likely play a role, but a more fundamental difference also exists: Zhang *et al.* derived their ES cells from *Bend3*-/- embryos, therefore BEND3 was never present in the ES cells. In contrast, we deleted *Bend3* in ES cells that previously expressed the gene. Therefore, the results of Zhang *et al.* could not discriminate whether BEND3 is involved in bivalent domain establishment, maintenance, or both. Our results establish unequivocally that BEND3 is required for bivalent genes to maintain their poised state, yet they do not rule out an additional role in establishing these domains.

Our ChIP-seq data show that a major determinant of BEND3 distribution on genomic DNA is the presence of consensus sites, with the sequence CCCACGCG. About 70% of all BEND3 peaks contain one or more such sites, and 20% of all BEND3 peaks contain three or more. Our findings are fully consistent with two recent papers showing that the BEN domain 4 of BEND3 recognizes the sequence CCACGC (13), but they differ from another study in which BEND3 was proposed to bind G-quadruplexes (10). The fact that only 10% of all bivalent promoters are regulated by BEND3 is likely directly linked to the presence or absence of the BEND3 consensus sites, but other contributing factors cannot be ruled out. The consensus BEND3 binding motif contains two CpG sites that can potentially be subjected to cytosine DNA methylation. Our data show that bound motifs are unmethylated, while methylated motifs are not bound, similarly to the binding behavior of BANP, a protein related to BEND3 (38). Structurally, CpG methylation has been shown to impair BEND3 binding by blocking an arginine-to-cytosine contact (13,36), which is consistent with our ChIP-seq data.

DNA methylation, therefore, influences BEND3 binding and is conversely regulated by BEND3. Indeed, a striking finding we report is that mouse ES cells lacking BEND3 have decreased DNA hydroxymethylation, combined with increased DNA methylation, suggesting that TET activity is lower in the absence of BEND3. In agreement with this possibility, while the DNA hypermethylation of *Bend3* KO cells is global across the genome, it is especially marked at certain regions such as enhancers and CpG shelves, which are normally protected against de novo methylation by the TET enzymes (39–44). We have ruled out a direct transcriptional downregulation of TET1, TET2, or TET3 as the cause for decreased TET activity. However, the TET enzymes are critically dependent on alpha-ketoglutarate (alpha-KG) for catalysis, and the promoters of the two key genes for alpha-KG production, *Idh1* and *Idh2*, are bound and activated by BEND3, therefore we speculate that lower levels of alpha-KG contribute to decreased TET activity in the mutant cells.

Many genes are differentially expressed upon BEND3 removal, even though they are not directly bound. It is likely that some of these indirect targets become repressed because their promoter or enhancer gains abnormal levels of DNA methylation upon BEND3 removal. We and others (13) find that loss of BEND3 leads to a decrease of H3K27me3 at BEND3 sites. This could be explained by a direct effect of BEND3 on H3K27 methylation, such as the recruitment of NuRD, which deacetylates H3K27 as a necessary step towards methylation (10). However, CpG methylation is antagonistic with H3K27 methylation, and our data suggest that increased cytosine methylation in the *Bend3* mutant cells could also contribute to the decreased H3K27me3 deposition. Future experiments will help address this possibility.

## Supplementary Material

gkad719_Supplemental_Files

## Data Availability

The accession number for the deep sequencing data reported in this paper are: GSE221710 (CRISPR screen, Mutational analysis) and GSE224077 (RNA-seq, WGBS, ChIP-seq, CUT&RUN-seq). The scripts used are available on Github: https://github.com/FumihitoMiura/Project-2 (permanent DOI: 10.6084/m9.figshare.23899266) and https://github.com/kirsho/DASH (permanent DOI: 10.6084/m9.figshare.23899254).

## Notes

Present address: Nikhil Gupta, Joint AstraZeneca-Cancer Research Horizons Functional Genomics Centre, Jeffrey Cheah Biomedical Centre, Cambridge, UK.
