# Supplementary material for: A genetic screen identifies BEND3 as a regulator of bivalent gene expression and global DNA methylation
Source: Nucleic Acids Res. 2023 Aug 31;51(19):10292–308. doi: 10.1093/nar/gkad719 (PMC10602864; doi:10.1093/nar/gkad719)

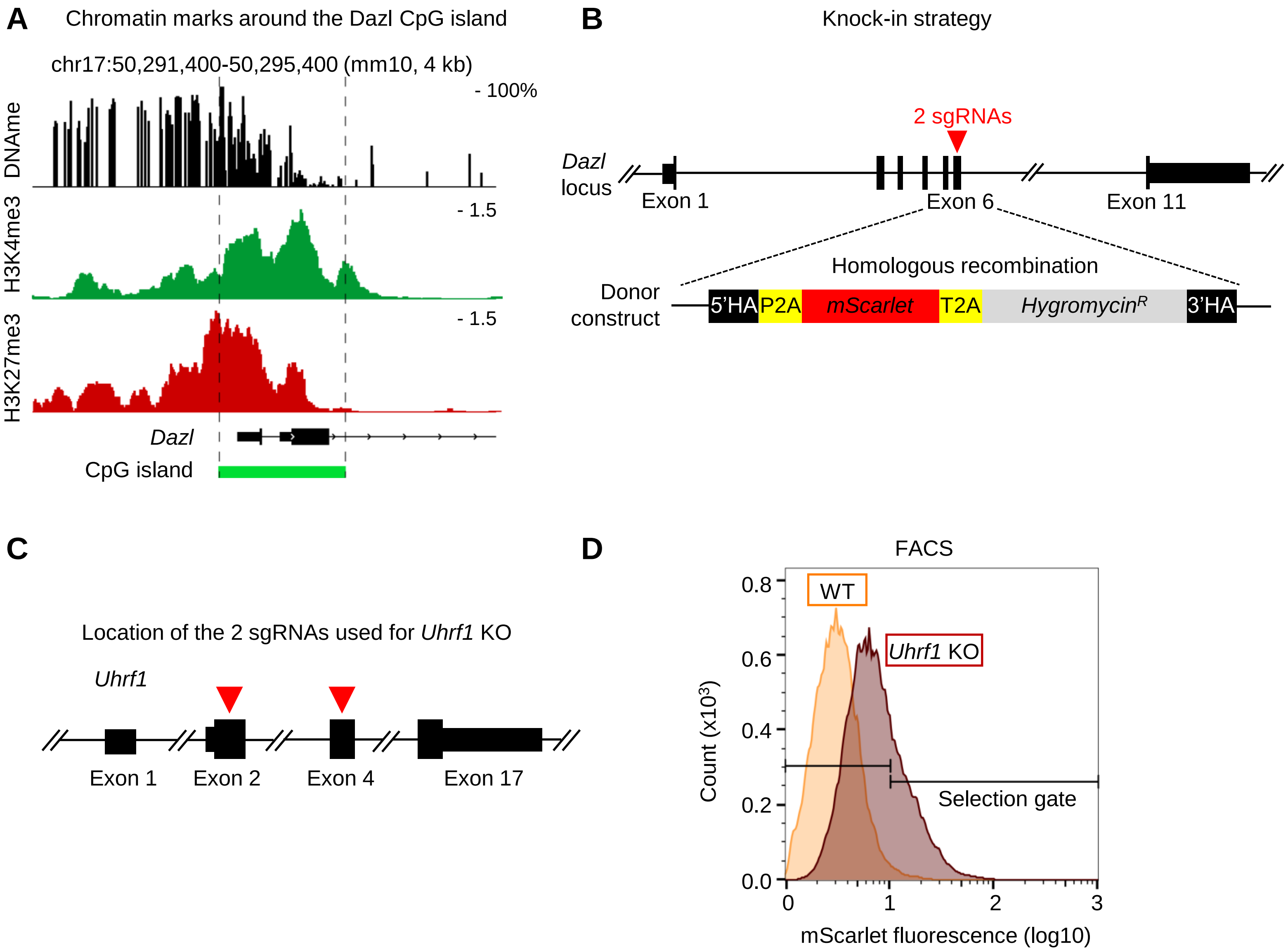

Figure S1: *Knock-in and knock-out strategies. .* (A) Chromatin marks surrounding the *Dazl* CpG island. The DNA methylation data is WGBS from our study. The H3K4me3 and H3K27me3 data are from (16) (GSE98149) (B) Two selectable markers, mScarlet and HygroR, were knocked in, in frame, in exon 6 of *Dazl.* (C) Position of the guide RNAs used to obtain a *Uhrf1* KO population*.* (D) FACS data showing the expression of mScarlet in WT and *Uhrf1* KO cells.

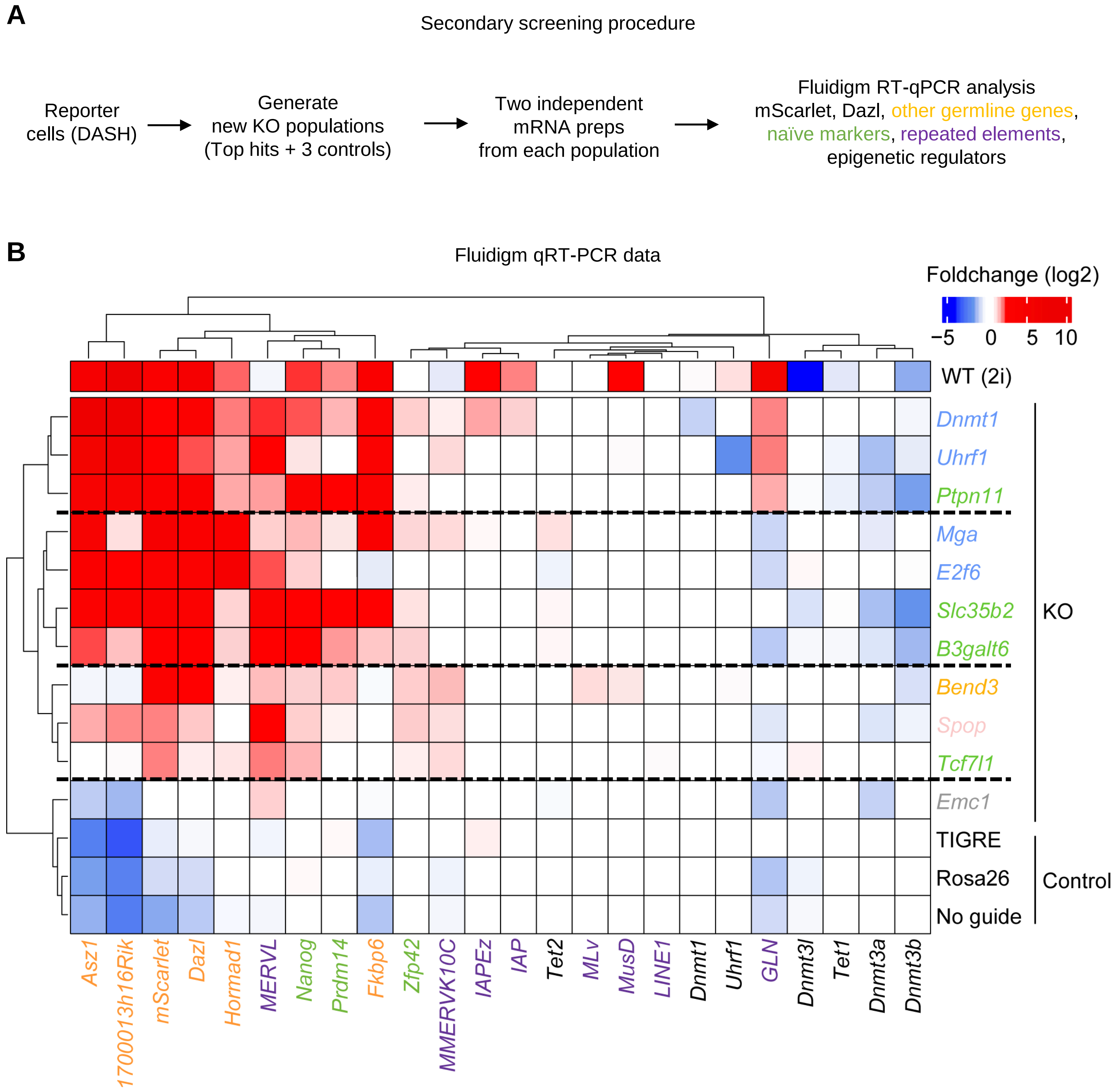

Figure S2*: Secondary screen design and results.* (A) Procedure followed for the secondary screen. (B) Results of the Fluidigm qRT-PCR analysis. Genes for which expression was measured are presented vertically, with the color code of panel A. The horizontal lines represent the cellular populations (Controls or KO) that were assayed. An unsupervised clustering was performed, and the clusters are separated by dashed lines for easier visualization.

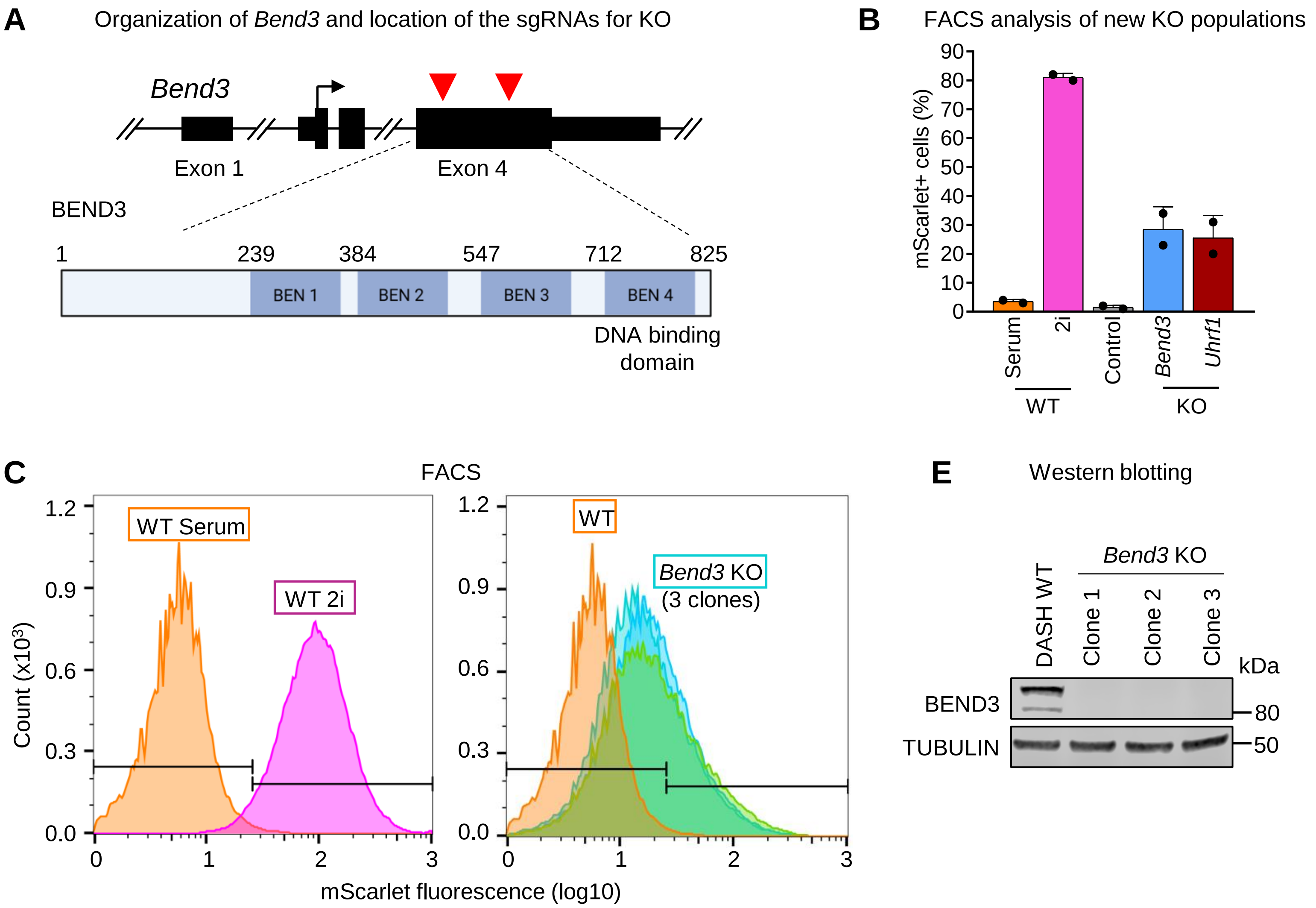

D

KO	ID	WT size (aa)	Clone no	Mutant protein	
				Allele 1	Allele 2
Bend3	NP_950193	825	1	p.V308_Y311del	
			2	p.V308Gfs*5	p.V308*
			3	p.V308_Y312del / p.P491Lfs*3	p.V308*

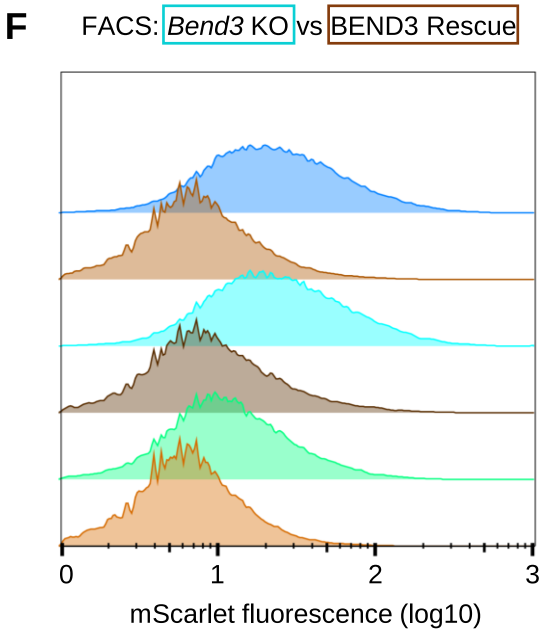

Figure S3 (preceding page): *Generation and analysis of the* Bend3 *KO mutations.* (A) Position of the sgRNAs used to generate *Bend3* KO populations and clones. (B) A FACS analysis on independent *Bend3* mutant populations validates the results of the initial screen. (C) Primary FACS data showing WT cells in serum (orange) and 2i medium (pink) in the left panel; control cells (gray), *Bend3* KO (blue) and *Uhrf1* KO (brown) in the middle panel; and the three individual *Bend3* mutant clones (dark blue, light blue, green), compared to WT cells (orange), in the right panel. (D) Nature of the mutations identified in the independent *Bend3* mutant clones, and their consequence at the protein level. (E) The *Bend3* mutant clones do not express detectable levels of BEND3 protein. (F) Primary FACS data showing the profiles for each of the three *Bend3* KO clones (dark blue, light blue, green) and their respective rescue populations, expressing V5-BEND3 (light brown, dark brown, orange).

Figure S4 (following page)*: Additional RNA-seq analyses.* (A) Heatmap showing the genes upregulated in *Bend3* KO cells (n=325), and genes downregulated in *Bend3* KO cells (n=635) (B) Overlap between the differentially expressed genes found in our study, and those reported by Zhang et al (CRA004815) (13). (C) Analysis of Transposable Element expression in our RNA-seq dataset. Left panel: *Bend3* KO cells compared to WT, right panel: WT cells in 2i compared to WT cells in serum. (D) Left panel: a GO-term analysis shows that developmental genes are enriched within genes differentially expressed in *Bend3* KO cells. Right panel: an illustration of some of these regulators.

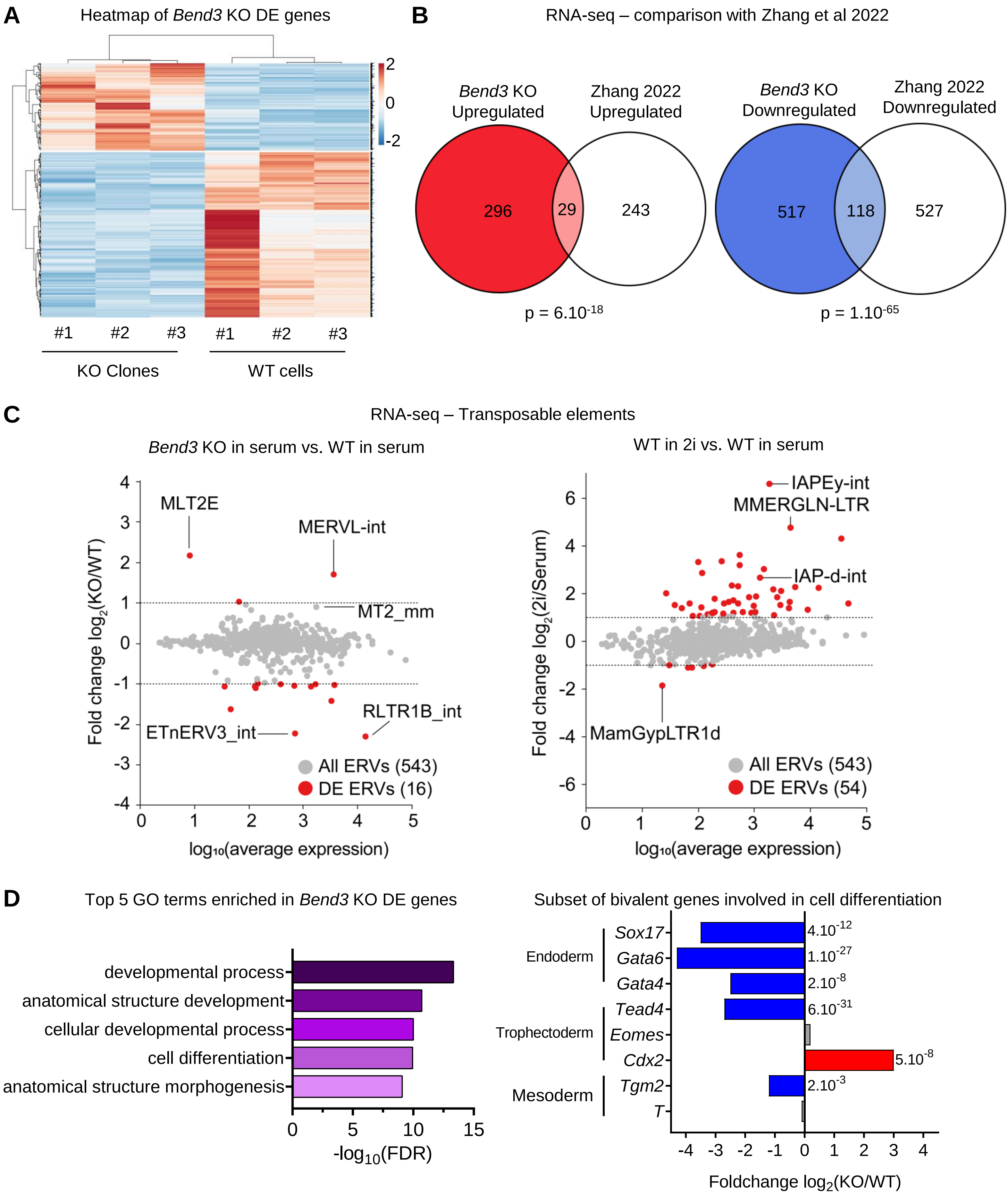
A
Heatmap of Bend3 KO DE genes
2
0
-2
#1
#2
#3
#1
#2
#3
KO Clones
WT cells
B
RNA-seq – comparison with Zhang et al 2022
Bend3 KO Upregulated
Zhang 2022 Upregulated
296
29
243
p = 6.10-18
Bend3 KO Downregulated
Zhang 2022 Downregulated
517
118
527
p = 1.10-65
C
RNA-seq – Transposable elements
Bend3 KO in serum vs. WT in serum
MLT2E
MERVL-int
MT2_mm
ETnERV3_int
RLTR1B_int
All ERVs (543)
DE ERVs (16)
Fold change log2(KO/WT)
log10(average expression)
WT in 2i vs. WT in serum
IAPEy-int
MMERGLN-LTR
IAP-d-int
MamGypLTR1d
All ERVs (543)
DE ERVs (54)
Fold change log2(2i/Serum)
log10(average expression)
D
Top 5 GO terms enriched in Bend3 KO DE genes
developmental process
anatomical structure development
cellular developmental process
cell differentiation
anatomical structure morphogenesis
0
5
10
15
-log10(FDR)
Subset of bivalent genes involved in cell differentiation
Endoderm
Sox17
Gata6
Gata4
Trophectoderm
Tead4
Eomes
Cdx2
Mesoderm
Tgm2
T
4.10-12
1.10-27
2.10-8
6.10-31
5.10-8
2.10-3
Foldchange log2(KO/WT)

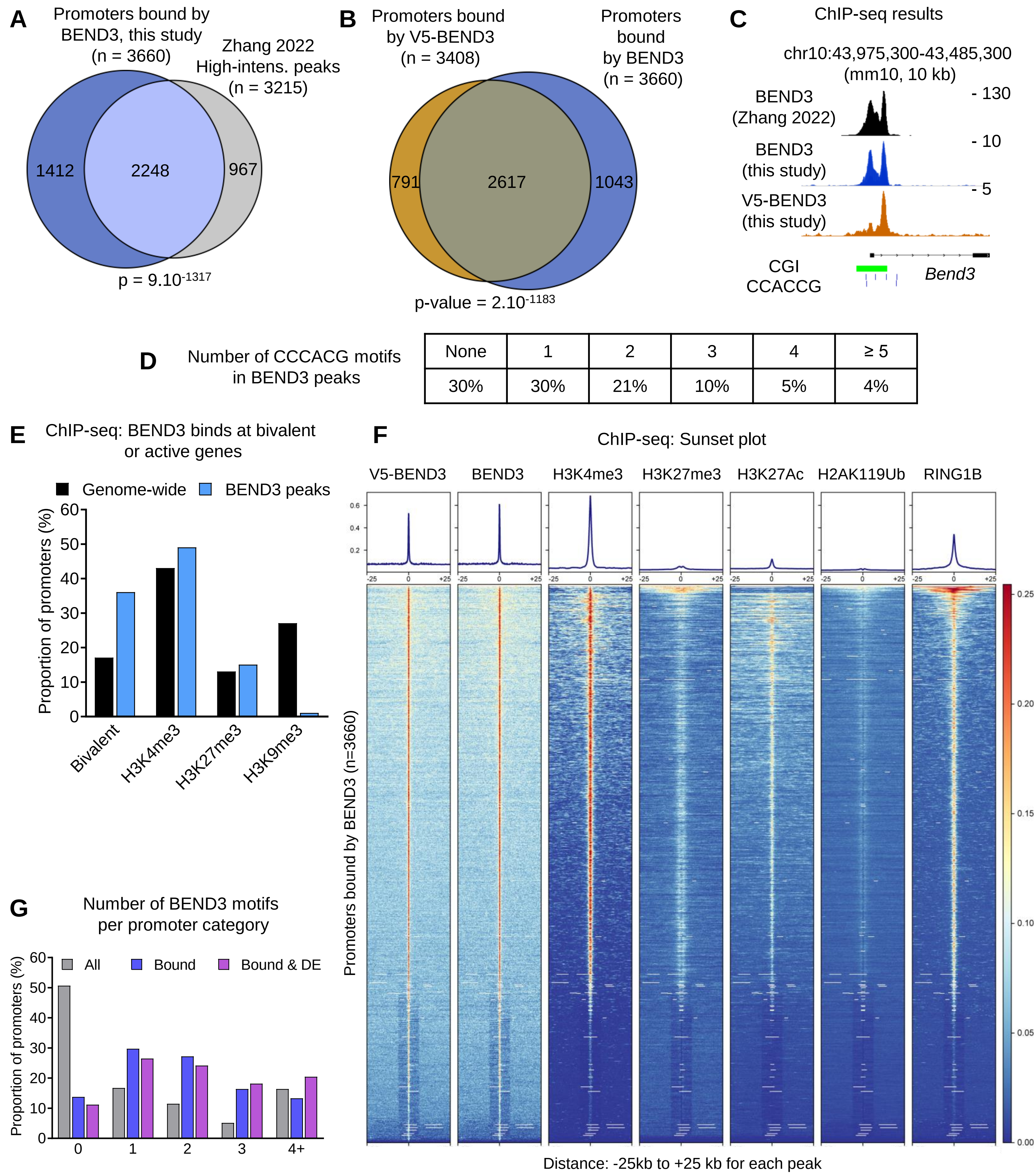

Figure S5: *Additional controls of the ChIP-seq data*. (A) Comparison of our BEND3 ChIP-seq with the dataset of Zhang et al. (B) Overlap of the ChIP-seq data obtained with antibodies against endogenous BEND3, or against the V5 tag. (C) Illustration of the similarity of the Zhang dataset and our dataset at the promoter of *Bend3*. (D) Number of consensus motifs in the BEND3-bound peaks (our dataset) (E) BEND3 binding is enriched on bivalent promoters, and depleted from H3K9me3-marked promoters. (F) Heatmap comparing the ChIP-seq profiles in mES cells of V5-BEND3 and BEND3 (both from our work), H3K4me3, H3K27me3, H3K27Ac (GSE99530) (35), H2AK119Ub (GSE126864) (45), and RING1B (GSE72164) (46). (G) Number of BEND3 consensus binding sites per promoter category. DE: Differentially Expressed in the KO.

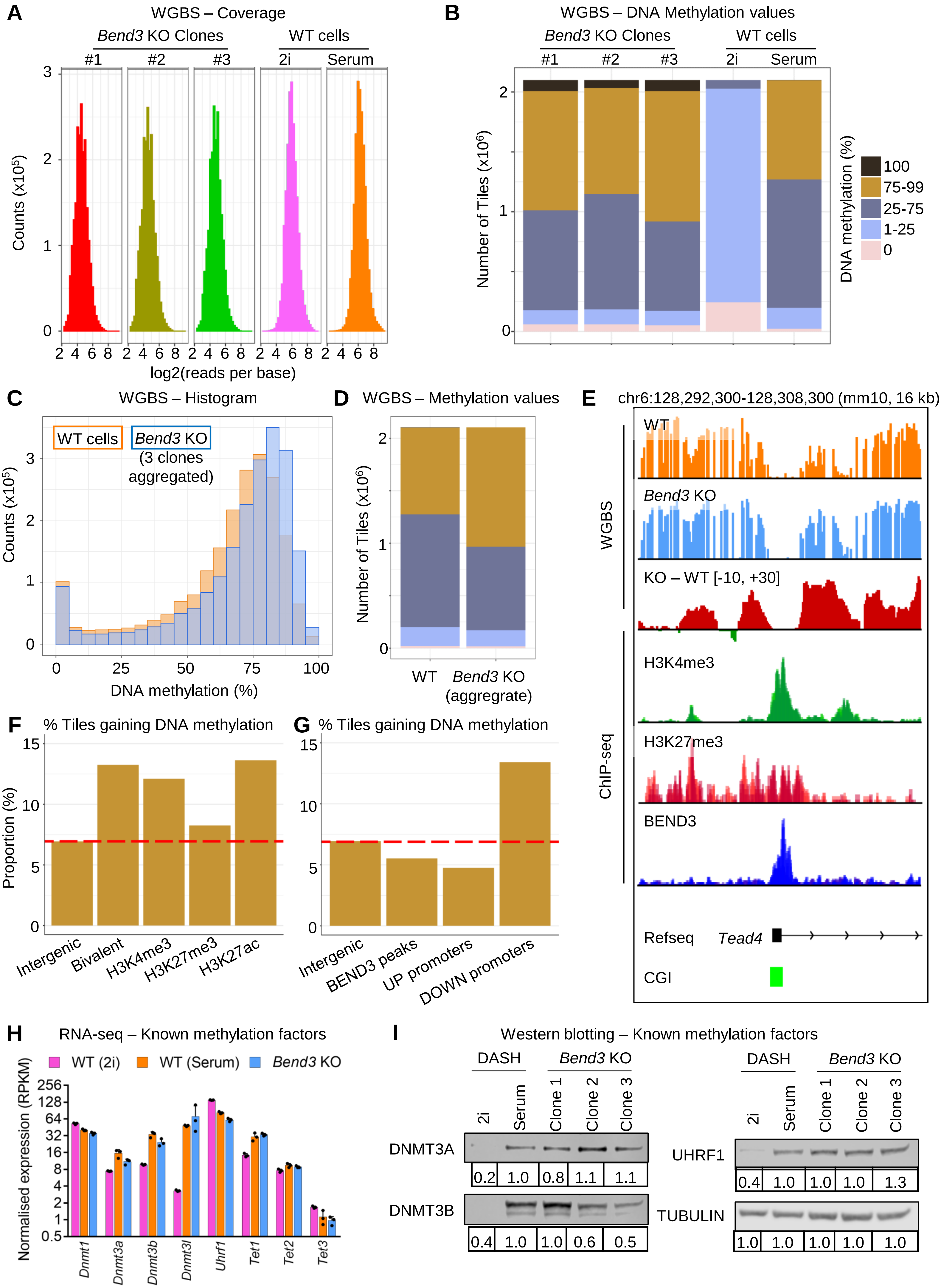
A
WGBS – Coverage
Bend3 KO Clones
WT cells
#1
#2
#3
2i
Serum
Counts (x10^5)
log2(reads per base)
B
WGBS – DNA Methylation values
Bend3 KO Clones
WT cells
#1
#2
#3
2i
Serum
Number of Tiles (x10^6)
DNA methylation (%)
100
75-99
25-75
1-25
0
C
WGBS – Histogram
WT cells
Bend3 KO
(3 clones
aggregated)
Counts (x10^5)
DNA methylation (%)
D
WGBS – Methylation values
Number of Tiles (x10^6)
WT
Bend3 KO
(aggregate)
E
chr6:128,292,300-128,308,300 (mm10, 16 kb)
WGBS
WT
Bend3 KO
KO – WT [-10, +30]
ChIP-seq
H3K4me3
H3K27me3
BEND3
Refseq
Tead4
CGI
F
% Tiles gaining DNA methylation
Proportion (%)
Intergenic
Bivalent
H3K4me3
H3K27me3
H3K27ac
G
% Tiles gaining DNA methylation
Intergenic
BEND3 peaks
UP promoters
DOWN promoters
H
RNA-seq – Known methylation factors
WT (2i)
WT (Serum)
Bend3 KO
Normalised expression (RPKM)
Dnmt1
Dnmt3a
Dnmt3b
Dnmt3l
Uhrf1
Tet1
Tet2
Tet3
I
Western blotting – Known methylation factors
DASH
Bend3 KO
2i
Serum
Clone 1
Clone 2
Clone 3
DNMT3A
0.2 1.0 0.8 1.1 1.1
DNMT3B
0.4 1.0 1.0 0.6 0.5
UHRF1
0.4 1.0 1.0 1.0 1.3
TUBULIN
1.0 1.0 1.0 1.0 1.0

Figure S6 (preceding page): *Additional WGBS analyses; DNA methylation gain in* Bend3 *KO is not associated with* DNMTs overexpression. (A) WGBS coverage data. The three individual *Bend3* KO clones are shown on the left, WT cells grown in 2i are shown in pink, and WT cells grown in serum are shown in orange. (B) Absolute number of tiles in the indicated methylation categories. (C) Distribution of DNA methylation values in WT (orange) vs. *Bend3* KO cells (blue), both grown in serum. (D) Absolute number of tiles in the indicated methylation categories. Same color code as in panel B. (E) Example of methylation gains around the CGI of *Tead4*, a bivalent gene that is bound by BEND3 and downregulated upon *Bend3* KO. (F) Percentage of tiles hypermethylated in the indicated categories. (G) Percentage of tiles hypermethylated in the indicated categories. (H) mRNA level for the indicated epigenetic regulators in the different cell types. (I) Western blotting and quantification in the indicated cellular backgrounds.

Figure S7 (following page): Additional data related to chromatin changes in *Bend3* mutant cells.
(A) Contingency tables between chromatin marks in DE genes. The most significant dependency is between BEND3 binding and H3K27me3 alteration in DE genes. p-value: chi-square tests. (B) Illustration of the WGBS, ChIP-seq, CUT&RUN, and RNA-seq data integration at *Dazl*. In *Bend3* KO cells, the *Dazl* promoter loses DNA methylation and H3K27me3. DMRs: tiles that gain DNA methylation (in black) or lose DNA methylation (in white). The absolute variations of methylation (gain/loss) are indicated. Red rectangle: H3K27me3 peak as detected in the CUT&RUN experiment on WT cells. (C) As in panel B but for the *Col4a1/2* promoter, which gains H3K27me3 and DNA methylation upon *Bend3* KO. (D) As in panel B but for *Cgas*, a bivalent gene. Upon *Bend3* KO, H3K27me3 is lost from the BEND3-binding CGI and neighboring regions, and gene expression is induced. (E) As in panel B but for *Magohb*. This promoter is not directly bound by BEND3; upon *Bend3* deletion it loses H3K27me3 and gains DNA methylation, while transcription is downregulated.

Supplementary Data – Reference list

45. Turberfield,A.H., Kondo,T., Nakayama,M., Koseki,Y., King,H.W., Koseki,H. and Klose,R.J. (2019) KDM2 proteins constrain transcription from CpG island gene promoters independently of their histone demethylase activity. *Nucleic Acids Res*, **47**, 9005–9023.

46. Joshi,O., Wang,S.-Y., Kuznetsova,T., Atlasi,Y., Peng,T., Fabre,P.J., Habibi,E., Shaik,J., Saeed,S., Handoko,L., *et al.* (2015) Dynamic Reorganization of Extremely Long-Range Promoter-Promoter Interactions between Two States of Pluripotency. *Cell Stem Cell*, **17**, 748–757.

A WGBS — Contingency tables on DE gene promoters — BEND3 ChIP-seq

WGBS (rows: CUT&RUN)

p = 6.10^-3	Hyper-methylation	Hypo-methylation	No change	
H3K27me3 lost	139	14	178	331
H3K27me3 gained	40	2	105	147
No change	188	13	281	482
	367	29	564	960

BEND3 ChIP-seq (rows: CUT&RUN)

p = 8.10^-13	Bound	Not Bound	
H3K27me3 lost	98	233	331
H3K27me3 gained	56	91	147
No change	62	420	482
	216	744	960

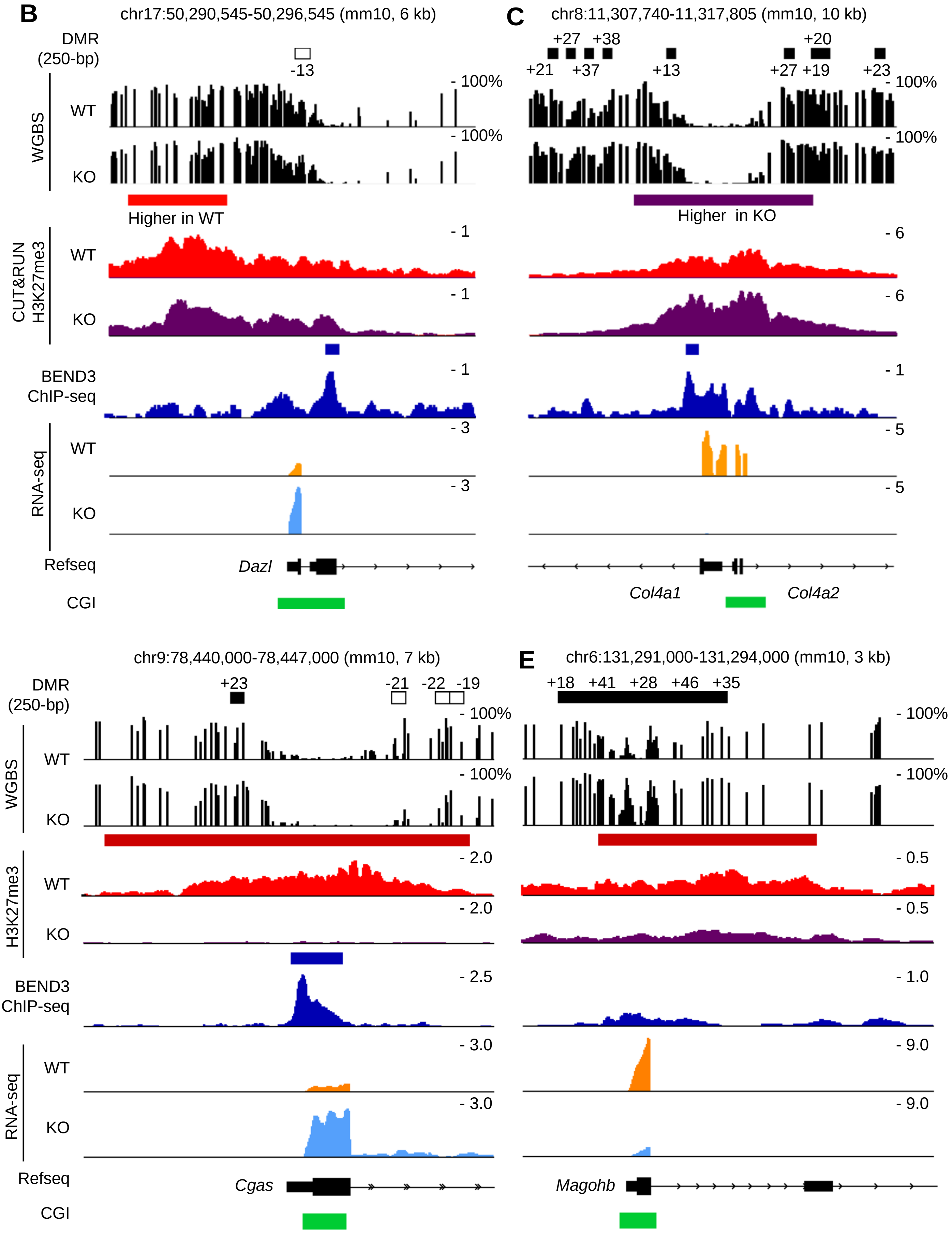

Supplement: gkad719_Supplemental_Files [file gkad719_supplemental_files.zip › Supplementary Data.pdf]
